# Bayesian model reduction and empirical Bayes for group (DCM) studies

**DOI:** 10.1016/j.neuroimage.2015.11.015

**Published:** 2016-03

**Authors:** Karl J. Friston, Vladimir Litvak, Ashwini Oswal, Adeel Razi, Klaas E. Stephan, Bernadette C.M. van Wijk, Gabriel Ziegler, Peter Zeidman

**Affiliations:** a The Wellcome Trust Centre for Neuroimaging, UCL, 12 Queen Square, London, UK; b Department of Electronic Engineering, NED University of Engineering & Technology, Karachi, Pakistan; c Translational Neuromodeling Unit (TNU), Institute for Biomedical Engineering, University of Zurich and ETH Zurich, Switzerland

**Keywords:** Empirical Bayes, Random effects, Fixed effects, Dynamic causal modelling, Classification, Bayesian model reduction, Hierarchical modelling

## Abstract

This technical note describes some Bayesian procedures for the analysis of group studies that use nonlinear models at the first (within-subject) level – e.g., dynamic causal models – and linear models at subsequent (between-subject) levels. Its focus is on using Bayesian model reduction to finesse the inversion of multiple models of a single dataset or a single (hierarchical or empirical Bayes) model of multiple datasets. These applications of Bayesian model reduction allow one to consider parametric random effects and make inferences about group effects very efficiently (in a few seconds). We provide the relatively straightforward theoretical background to these procedures and illustrate their application using a worked example. This example uses a simulated mismatch negativity study of schizophrenia. We illustrate the robustness of Bayesian model reduction to violations of the (commonly used) Laplace assumption in dynamic causal modelling and show how its recursive application can facilitate both classical and Bayesian inference about group differences. Finally, we consider the application of these empirical Bayesian procedures to classification and prediction.

## Highlights

• We describe a novel scheme for inverting non-linear models (e.g. DCMs) within subjects and linear models at the group level
• We demonstrate this scheme is more robust to violations of the (commonly used) Laplace assumption than the standard approach
• We validate the approach using a simulated mismatch negativity study of schizophrenia
• We demonstrate the application of this scheme to classification and prediction of group membership

## Introduction

This paper introduces some potentially useful procedures for the analysis of data from group studies using nonlinear models; for example, dynamic causal models of neurophysiological timeseries. Its key contribution is to finesse the problems that attend the inversion or fitting of hierarchical models in a nonlinear setting. This is achieved by using *Bayesian model reduction* that allows one to compute posterior densities over model parameters, under new prior densities, without explicitly inverting the model again. For example, one can invert a nonlinear (dynamic causal) model for each subject in a group and then evaluate the posterior density over group effects, using the posterior densities over parameters from the single-subject inversions. This application can be regarded as a generalisation of the standard summary statistic approach; however, instead of just using point estimators as summaries of first (within-subject) level effects, one can take the full posterior density to the second (between-subject) level. Furthermore, this *empirical Bayes* procedure can be applied to any model inversion scheme that furnishes posterior densities, which can be summarised with a multivariate Gaussian distribution.

Bayesian model reduction refers to the Bayesian inversion and comparison of models that are reduced (or restricted) forms of a full (or parent) model. It can be applied whenever models can be specified in terms of (reduced) prior densities. A common example would be switching off a parameter in a full model by setting its prior mean and variance to zero. The important aspect of Bayesian model reduction is that models differ only in their priors, which means that the posterior of a reduced model can be derived from the posterior of the full model. In this paper, we will use Bayesian model reduction to evaluate empirical priors to provide an *empirical Bayesian model reduction* scheme.

Empirical Bayes refers to the Bayesian inversion or fitting of hierarchical models. In hierarchical models, constraints on the posterior density over model parameters at any given level are provided by the level above. These constraints are called *empirical priors* because they are informed by empirical data. In this paper, we will consider an empirical Bayesian approach to any hierarchical model that can be expressed in terms of an arbitrary (nonlinear) model at the first level and a standard (parametric) empirical Bayesian (PEB) model at higher levels (Efron and Morris, 1973, Kass and Steffey, 1989). In other words, if the parameters of a nonlinear model of subject-specific data are generated by adding random (Gaussian) effects to group means, then the procedures of this paper can be applied. Crucially, these procedures are very efficient because each hierarchical level of the model requires only the posterior density over the parameters of the level below. This means, one can invert deep hierarchical models without having to revisit lower levels. This aspect of the scheme rests on Bayesian model reduction, a procedure that we have previously described in the context of *post hoc* model optimisation and discovery (Friston and Penny, 2011, Friston et al., 2011, Rosa et al., 2012). Here, it is put to work in the context of empirical Bayes and, as we will see later, evaluating predictive posterior densities for classification.

We envisage empirical Bayesian model reduction will be applied primarily to group Dynamic Causal Modelling (DCM) studies, where subjects are assigned to groups according to factors such as behaviour, diagnosis or genetics (e.g. Bernal-Casas et al., 2012). However, the ideas presented here are not limited to DCM. They can be applied to any nonlinear model and, interestingly, any inversion scheme at the first (within-subject) level. This may be particularly important for harnessing the computational investment of schemes that use stochastic methods to evaluate first level posteriors (Sengupta et al., 2016). Bayesian model reduction resolves (or at least frames) a number of issues in the inversion and interpretation of group DCM studies. Consequently, we will take the opportunity to illustrate some of these issues using a worked example. These include the problem of local maxima when evaluating different models for Bayesian model comparison—and the fundamental distinction between random (between-subject) effects at the level of models and their parameters. In contrast to our previous treatment of random model effects at the between-subject level (Stephan et al., 2009), this paper considers random parameter effects in the setting of parametric empirical Bayes. We will also look at the fundamental difference between classical and Bayesian inference about group effects. Finally, we will briefly consider Bayesian classification of single subjects and touch on (leave-one-out) cross validation.

This paper comprises four sections. The first reviews Bayesian model reduction and introduces its application in a hierarchical or empirical Bayesian setting. This section reviews the basic theory, which generalises conventional approaches to random effects modelling. The second section applies the theory of the first to group studies, providing specific expressions for the procedures used in subsequent sections. The third section considers Bayesian model reduction using a worked example based on a (simulated) DCM study of mismatch negativity. The focus of this section is the utility of Bayesian model reduction in finessing (e.g., local maxima) problems that are often encountered when inverting nonlinear models. We will see that Bayesian model reduction provides more robust estimates of posterior probabilities than fitting models to the data separately, because it is less susceptible to violations of (e.g., Laplace) assumptions. This application of Bayesian model reduction provides Bayesian model averages that could be used for classical inference with the standard summary statistic approach, which we illustrate using canonical covariates analysis. However, one can go further in terms of model comparison and classification using empirical Bayesian model reduction. The last section revisits the worked example to illustrate model comparison and averaging at the second (between-subject) level. Our focus here is on inference about group effects and classification using the posterior predictive density afforded by empirical priors. The worked example was chosen to be representative of real DCM studies—so that the procedures could be illustrated in a pragmatic way. We will therefore refer to specific (Matlab) routines that implement the procedures. These routines are part of the academic SPM software available from http://www.fil.ion.ucl.ac.uk/spm.

## Methods and theory

### Bayesian model reduction

Bayesian model reduction refers to the Bayesian inversion of reduced models using only the posterior densities of a full model. Bayesian model reduction provides an efficient way to invert large numbers of (reduced) models, following the (usually computationally expensive) inversion of a full model. Consider a generative model that is specified in terms of its likelihood and priors. For example, models with additive Gaussian noise have the following form:

(1) $$\begin{array}{c} \ln p\left(y,\theta |m\right)=\ln p\left(y|\theta ,m\right)+\ln p\left(\theta |m\right)\\ p\left(y|\theta ,m\right)=N\left(\Gamma \left(\theta \right),\Sigma \left(\theta \right)\right)\\ p\left(\theta |m\right)=N\left(\eta ,\Sigma \right) \end{array}$$

Here, *Γ*(*θ*) is a possibly nonlinear mapping from the parameters of a model to the predicted response *y*. Gaussian assumptions about observation noise, with a parameterised covariance *Σ*(*θ*), define the likelihood model that, when equipped with (Gaussian) priors, specifies the generative model. The generative model provides a probabilistic mapping from model parameters to observed data. Inference corresponds to the inversion of this mapping; from data to parameters. Usually, this inversion uses some form of approximate Bayesian inference.

Approximate Bayesian inference can always be cast as maximising the (negative) variational free energy with respect to the sufficient statistics $\tilde{q}$ of an approximate posterior $q\left(\theta |\tilde{q}\right)$: see (Roweis and Ghahramani, 1999, Friston, 2008) for a fuller discussion. In this paper, a tilde (~) denotes the set of sufficient statistics of the prior $\tilde{p}$ and posterior $\tilde{q}$. Under the Laplace assumption (used throughout this work), the sufficient statistics correspond to the mean and covariance of each density. Using $\tilde{p}=\left(\eta ,\Sigma \right)$ for the sufficient statistics of the prior, approximate Bayesian inference therefore corresponds to the optimisation problem:

(2) $$\begin{array}{c} {\tilde{q}}^{*}=\arg\max_{\overset{⌢}{q}}F\left(\tilde{p},\tilde{q}\right)\\ F\left(\tilde{p},\tilde{q}\right)=\underset{\mathrm{accuracy}}{\underset{︸}{E_{q}\left[\ln p\left(y|\theta \right)\right]}}-\underset{\mathrm{complexity}}{\underset{︸}{D_{KL}\left[q\left(\theta |\tilde{q}\right)||p\left(\theta |\tilde{p}\right)\right]}} \end{array}$$

Here, we have expressed the free energy in terms of accuracy (first term) and complexity (second term), which is the Kullback–Leibler divergence between the (approximate) posterior and prior. Usually, this optimisation would proceed using a Fisher scoring scheme or related gradient ascent: see (Friston et al., 2007) and the appendix for details. After the negative free energy has been maximised the following approximate equalities provide an estimate of the posterior density over unknown model parameters and the log evidence or (marginal) likelihood of the model itself:

(3) $$\begin{array}{c} q\left(\theta |{\tilde{q}}^{*}\right)\approx p\left(\theta |y,\tilde{p}\right)\\ F\left(\tilde{p},{\tilde{q}}^{*}\right)\approx \ln p\left(y|\tilde{p}\right) \end{array}$$

By expressing the free energy as a function of the sufficient statistics of the prior and approximate posterior, it can be seen that the free energy depends on the prior, which in turn, specifies our beliefs about a model.

Now, say we wanted to estimate the posterior under a new model after eliminating some parameters to produce a reduced model. This is commonplace in classical statistics and corresponds to evaluating the treatment and residual sum of squares for a new contrast of parameters. Exactly the same idea can be applied to Bayesian inference. This rests upon the definition of a reduced model as a likelihood model with reduced priors. Consider Bayes rule replicated for reduced and full models (*m*_*R*_, *m*_*F*_):

(4) $$\begin{array}{ccc} \begin{array}{l} p\left(\theta |y,m_{R}\right)=\frac{p\left(y|\theta ,m_{R}\right)p\left(\theta |m_{R}\right)}{p\left(y|m_{R}\right)}\\ p\left(\theta |y,m_{F}\right)=\frac{p\left(y|\theta ,m_{F}\right)p\left(\theta |m_{F}\right)}{p\left(y|m_{F}\right)} \end{array} & \Leftrightarrow & \begin{array}{l} p\left(\theta |y,m_{R}\right)p\left(y|m_{R}\right)=p\left(y|\theta ,m_{R}\right)p\left(\theta |m_{R}\right)\\ p\left(\theta |y,m_{F}\right)p\left(y|m_{F}\right)=p\left(y|\theta ,m_{F}\right)p\left(\theta |m_{F}\right) \end{array} \end{array}$$

Crucially, if the models differ only in terms of their priors, then the likelihoods are identical and Eq. (4) can be simplified:

(5) $$\begin{array}{l} p\left(y|\theta ,m_{R}\right)=p\left(y|\theta ,m_{F}\right)\Rightarrow \\ \frac{p\left(\theta |y,m_{R}\right)p\left(y|m_{R}\right)}{p\left(\theta |y,m_{F}\right)p\left(y|m_{F}\right)}=\frac{p\left(\theta |m_{R}\right)}{p\left(\theta |m_{F}\right)} \end{array}$$

Here, we have expressed Bayes rule in terms of a posterior odds ratio, so that the likelihoods cancel. From Eq. (5) we can derive two quantities of interest: by re-arrangement we get the posterior distribution over the parameters of the reduced model, and by integrating over the parameters, we get the evidence ratio of the reduced and full models (where the left hand side of the reduced posterior integrates to unity):

(6) $$\begin{array}{l} p\left(\theta |y,m_{R}\right)=p\left(\theta |y,m_{F}\right)\frac{p\left(\theta |m_{R}\right)}{p\left(\theta |m_{F}\right)}\frac{p\left(y|m_{F}\right)}{p\left(y|m_{R}\right)}\Rightarrow \\ \int p\left(\theta |y,m_{F}\right)\frac{p\left(\theta |m_{R}\right)}{p\left(\theta |m_{F}\right)}\frac{p\left(y|m_{F}\right)}{p\left(y|m_{R}\right)}d\theta =1\Rightarrow \frac{p\left(y|m_{R}\right)}{p\left(y|m_{F}\right)}=\int p\left(\theta |y,m_{F}\right)\frac{p\left(\theta |m_{R}\right)}{p\left(\theta |m_{F}\right)}d\theta \end{array}$$

Substituting the approximate values for the model posterior $q\left(\theta |{\tilde{q}}^{*}\right)$ and model evidence $F\left(\tilde{p},{\tilde{q}}^{*}\right)$ from Eq. (3) and replacing models (*m*_*R*_, *m*_*F*_) with the sufficient statistics of their definitive priors $\left({\tilde{p}}_{R},{\tilde{p}}_{F}\right)$ we get:

(7) $$\begin{array}{c} q\left(\theta |{\tilde{q}}_{R}^{*}\right)\approx q\left(\theta |{\tilde{q}}_{F}^{*}\right)\frac{p\left(\theta |{\tilde{p}}_{R}\right)}{p\left(\theta |{\tilde{p}}_{F}\right)}\frac{p\left(y|{\tilde{p}}_{F}\right)}{p\left(y|{\tilde{p}}_{R}\right)}\\ F\left({\tilde{p}}_{R},{\tilde{q}}_{R}^{*}\right)\approx \ln\int q\left(\theta |{\tilde{q}}_{F}^{*}\right)\frac{p\left(\theta |{\tilde{p}}_{R}\right)}{p\left(\theta |{\tilde{p}}_{F}\right)}d\theta +F\left({\tilde{p}}_{F},{\tilde{q}}_{F}^{*}\right)\\ =F\left({\tilde{p}}_{R}|{\tilde{p}}_{F},{\tilde{q}}_{F}^{*}\right) \end{array}$$

These (approximate) equalities mean one can evaluate the posterior and evidence of any reduced model, given the posteriors of the full model. In other words, $F\left({\tilde{p}}_{R},{\tilde{q}}_{R}^{*}\right)=F\left({\tilde{p}}_{R}|{\tilde{p}}_{F},{\tilde{q}}_{F}^{*}\right)$ allows us to skip the optimization of ${\tilde{q}}_{R}$ and use the optimised posterior of the full model to compute the evidence (and posterior) of the reduced model directly. These computations can be performed quickly and efficiently, using the equalities in the next section (see Eq. (8)). The meaning of reduced can be seen clearly in the above expressions: a reduced model is one in which the prior odds ratio is well defined over all values of the parameters. In other words, we require $p\left(\theta |{\tilde{p}}_{F}\right)>0$ when $p\left(\theta |{\tilde{p}}_{R}\right)>0$ but not *vice versa*. As its name implies, Bayesian model reduction can only be used to compare models when all models of interest can be cast as reduced forms of a full model; in other words, the full model must contain all the parameters of any model that will be entertained. This means one cannot compare models that have a completely different form (e.g., conductance and convolution based neural mass models for electrophysiology). However, in practice, most model comparisons tend to be framed in terms of models with and without key (sets of) parameters.

### Bayesian model reduction with variational Laplace

Variational Laplace corresponds to approximate Bayesian inference when assuming the approximate posterior $q\left(\theta |m_{F}\right)=N\left(\mu ,C\right)$ is Gaussian. Under this Laplace assumption, the reduced forms of the approximate posterior and free energy have simple forms (see Friston and Penny, 2011):

(8) $$\begin{array}{c} p\left(\theta |{\tilde{p}}_{F}\right)=N\left(\eta_{F},\Sigma_{F}\right)\\ p\left(\theta |{\tilde{p}}_{R}\right)=N\left(\eta_{R},\Sigma_{R}\right)\\ q\left(\theta |{\tilde{p}}_{R}\right)=N\left(\mu_{R},C_{R}\right)\\ P_{R}=P_{F}+\Pi_{R}-\Pi_{F}\\ \mu_{R}=C_{R}\left(P_{F}\mu_{F}+\Pi_{R}\eta_{R}-\Pi_{F}\eta_{F}\right)\\ F\left({\tilde{p}}_{R},{\tilde{q}}_{R}^{*}\right)=F\left({\tilde{p}}_{R}|{\tilde{p}}_{F},{\tilde{q}}_{F}^{*}\right)\\ =\frac{1}{2}\ln|\Pi_{R}P_{F}C_{R}\Sigma_{F}|-\frac{1}{2}\left(\mu_{F}^{T}P_{F}\mu_{F}+\eta_{R}^{T}\Pi_{R}\eta_{R}-\eta_{F}^{T}\Pi_{F}\eta_{F}-\mu_{R}^{T}P_{R}\mu_{R}\right)+F\left({\tilde{p}}_{F},{\tilde{q}}_{F}^{*}\right) \end{array}$$

Here *Π* and *Σ* are the prior precision and covariance respectively, while *P* and *C* are the corresponding posterior precision and covariance. This equation is derived by substituting Gaussian forms for the probability density functions into Eq. (7); more details can be found in Friston and Penny (2011). Note that when a parameter is removed from the model, by shrinking its prior variance to zero, the prior and posterior moments become the same and the parameter no longer contributes to the reduced free-energy. Effectively, Eq. (8) allows us to score any reduced model or prior in terms of a reduced free energy, while directly evaluating the posterior over its parameters.

Although we have presented Bayesian model reduction in a general way, we will be applying it in several distinct contexts. The most obvious and simplest application is Bayesian model comparison or selection. In this instance, the reduced models are specified by the user in terms of (reduced) priors that specify a model or hypothesis space. The second key application, which we consider next, is empirical Bayesian model reduction. In this case, a model can be reduced using hierarchical or empirical Bayesian constraints. Here, the full model has priors without hierarchical (e.g., between subject) constraints, while the reduced models have empirical priors. Notice that one can apply empirical Bayesian model reduction to any model—including models that have been previously reduced using Bayesian model reduction.

### Empirical Bayesian model reduction

Model reduction can be especially useful in hierarchical models, where the reduced prior is provided by an empirical prior. For example, consider the empirical Bayes or hierarchical Bayesian model:

(9) $$\begin{array}{c} \ln p\left(y,\theta^{\left(1\right)},\theta^{\left(2\right)}|m\right)=\ln p\left(y|\theta^{\left(1\right)},m\right)+\ln p\left(\theta^{\left(1\right)}|\theta^{\left(2\right)},m\right)+\ln p\left(\theta^{\left(2\right)}|m\right)\\ p\left(y|\theta^{\left(1\right)},m\right)=N\left(\Gamma^{\left(1\right)}\left(\theta^{\left(1\right)}\right),\Sigma \left(\theta^{\left(1\right)}\right)\right)\\ p\left(\theta^{\left(1\right)}|\theta^{\left(2\right)},m\right)=N\left(\Gamma^{\left(2\right)}\left(\theta^{\left(2\right)}\right),\Sigma \left(\theta^{\left(2\right)}\right)\right)\\ p\left(\theta^{\left(2\right)}|m\right)=N\left(\eta ,\Sigma \right) \end{array}$$

where *Γ*^(1)^ is a possibly nonlinear mapping from first level parameters to observations and *Γ*^(2)^ is a possibly nonlinear mapping from second to first level parameters (e.g., from group means to subject specific parameters). One can now express the inversion of this model in terms of free energies at the first and second levels. These free energies have exactly the same forms as in Eq. (2) (and can be decomposed in terms of accuracy and complexity):

(10) $$\begin{array}{c} {\tilde{q}}_{F}^{\left(1\right)}=\arg\max_{{\overset{⌢}{q}}^{\left(1\right)}}F^{\left(1\right)}\left({\tilde{p}}_{F},{\tilde{q}}^{\left(1\right)}\right)\\ {\tilde{q}}_{R}^{\left(1\right)}=\arg\max_{{\overset{⌢}{q}}^{\left(1\right)}}F^{\left(2\right)}\left({\tilde{p}}^{\left(2\right)},{\tilde{q}}^{\left(1\right)},{\tilde{q}}^{\left(2\right)}\right)\\ {\tilde{q}}^{\left(2\right)}=\arg\max_{{\overset{⌢}{q}}^{\left(2\right)}}F^{\left(2\right)}\left({\tilde{p}}^{\left(2\right)},{\tilde{q}}_{R}^{\left(1\right)},{\tilde{q}}^{\left(2\right)}\right)\\ F^{\left(1\right)}\left({\tilde{p}}^{\left(1\right)},{\tilde{q}}^{\left(1\right)}\right)=\underset{\mathrm{accuracy}}{\underbrace{E_{q^{\left(1\right)}}\left[\ln p\left(y|\theta^{\left(1\right)},m\right)\right]}}-\underset{1st\;\mathrm{level}\;\mathrm{complexity}}{\underset{︸}{D_{KL}\left[q\left(\theta^{\left(1\right)}|{\tilde{q}}^{\left(1\right)}\right)||p\left(\theta^{\left(1\right)}|{\tilde{p}}^{\left(1\right)}\right)\right]}}\\ F^{\left(2\right)}\left({\tilde{p}}^{\left(2\right)},{\tilde{q}}^{\left(1\right)},{\tilde{q}}^{\left(2\right)}\right)=\underset{\mathrm{accuracy}}{\underbrace{E_{{\tilde{q}}^{\left(1\right)}}\left[\ln p\left(y|\theta^{\left(1\right)},m\right)\right]}}-\underset{1st\;\mathrm{and}\;2nd\;\mathrm{evel}\;\mathrm{complexity}}{\underbrace{D_{KL}\left[q\left(\theta^{\left(1\right)}|{\tilde{q}}^{\left(1\right)}\right)q\left(\theta^{\left(2\right)}|{\tilde{q}}^{\left(2\right)}\right)||p\left(\theta^{\left(1\right)}|\theta^{\left(2\right)}\right)p\left(\theta^{\left(2\right)}|{\tilde{p}}^{\left(2\right)}\right)\right]]}}\\ =\underset{\mathrm{accuracy}}{\underbrace{E_{q^{\left(1\right)}}\left[\ln p\left(y|\theta^{\left(1\right)},m\right)\right]}}-\underset{1st\;\mathrm{level}\;\mathrm{complexity}}{\underbrace{E_{q^{\left(2\right)}}[D_{KL}\left[q\left(\theta^{\left(1\right)}|{\tilde{q}}^{\left(1\right)}\right)||p\left(\theta^{\left(1\right)}|{\tilde{p}}_{R}\right)\right]}}-\underset{2nd\;\mathrm{level}\;\mathrm{complexity}}{\underbrace{D_{KL}\left[q\left(\theta^{\left(2\right)}|{\tilde{q}}^{\left(2\right)}\right)||p\left(\theta^{\left(2\right)}|{\tilde{p}}^{\left(2\right)}\right)\right]}}\\ =\underset{\mathrm{accuracy}\;\mathrm{and}\;1st\;\mathrm{level}\;\mathrm{complexity}}{\underbrace{E_{q^{\left(2\right)}}\left[F^{\left(1\right)}\left({\tilde{p}}_{R},{\tilde{q}}^{\left(1\right)}\right)\right]}}-\underset{2nd\;\mathrm{level}\;\mathrm{complexity}}{\underbrace{D_{KL}\left[q\left(\theta^{\left(2\right)}|{\tilde{q}}^{\left(2\right)}\right)||p\left(\theta^{\left(2\right)}|{\tilde{p}}^{\left(2\right)}\right)\right]}}\\ {\tilde{p}}_{R}=\underset{\mathrm{empirical}\;\mathrm{prior}}{\underbrace{\left(\Gamma \left(\theta^{\left(2\right)}\right),\Sigma \left(\theta^{\left(2\right)}\right)\right)}}\\ F^{\left(2\right)}\left({\tilde{p}}^{\left(2\right)},{\tilde{q}}_{R}^{\left(1\right)},{\tilde{q}}^{\left(2\right)}\right)=\underset{\mathrm{accuracy}\;\mathrm{and}\;1st\;\mathrm{level}\;\mathrm{complexity}}{\underbrace{E_{q^{\left(2\right)}}\left[F^{\left(1\right)}\left({\tilde{p}}_{R}|{\tilde{p}}_{F},{\tilde{q}}_{F}^{\left(1\right)}\right)\right]}}-\underset{2nd\;\mathrm{level}\;\mathrm{complexity}}{\underbrace{D_{KL}\left[q\left(\theta^{\left(2\right)}|{\tilde{q}}^{\left(2\right)}\right)||p\left(\theta^{\left(2\right)}|{\tilde{p}}^{\left(2\right)}\right)\right]}} \end{array}$$

This free energy optimisation problem has been written in a way that clarifies the role of reduced free energy $F^{\left(1\right)}\left({\tilde{p}}_{R}|{\tilde{p}}_{F},{\tilde{q}}_{F}^{\left(1\right)}\right)$. Here, the (full) approximate posterior is evaluated in the usual way using relatively uninformative (full) priors. Following this, the approximate posterior over second level parameters can be computed from a (second level) free energy that comprises the expected (reduced) free energy from the first level and the complexity attributable to the posterior over second level parameters. Crucially, the reduced free energy is a function of the approximate posterior over second level parameters and the (known) approximate posterior over the first level parameters, under the full model. Effectively, this means the expected (reduced) free energy is the free energy that we would get if we replaced the full priors with the empirical priors afforded by the second level of the model. In short, we never need to actually optimise the first level posterior, when optimising the posterior at the second level.

An alternative perspective on this optimisation is that the reduced free energy function contains all the information that is necessary to optimise the parameters at the second level. This inversion scheme is fundamentally different from the standard empirical Bayes because it proceeds one level at a time. In other words, it would be possible to continue optimising the parameters at successively higher levels of deep hierarchical models, without re-estimating the approximate posteriors of lower levels. We will see examples of this later when we look at second level Bayesian model reduction and classification. We will also see that empirical Bayesian model reduction can be computationally very efficient, particularly when the first level involves the inversion of multiple datasets or subjects. In this setting, the first level contributes the sum of reduced free energy over subjects, because the posterior distributions are conditionally independent. This means one can accumulate evidence over subjects by inverting subject-specific models and test hypotheses at the between-subject level later, using the approximate posteriors.

### Summary

In summary, with a straightforward application of Bayes rule (Eq. (6)), one can express the posterior density of any (reduced) model in terms of the posterior of its parent or full model. This affords an efficient way to evaluate posterior densities under empirical priors; leading to the notion of hierarchical or empirical Bayesian model reduction. This form of hierarchical model inversion and comparison is interesting because it only requires the forward passing of the posterior density, from a lower level to a higher level, to generalise the standard summary statistical approach. In this generalisation, all the sufficient statistics of the posterior are passed to higher levels (as opposed to just passing the maximum likelihood or *a posteriori* parameter estimates). If we further assume the posterior at the lower level can be summarised with a Gaussian density (the Laplace assumption), then we have a very simple form for the reduced energy (Eq. (8); implemented in **spm_log_evidence.m**).

Although the treatment of this section may appear abstract, it generalises all parametric empirical Bayesian (PEB) approaches and classical random (or mixed) effect analyses of covariance. This generalisation is at two levels. First, by explicitly accommodating priors at each level of the hierarchical model we convert a classical random effects model into a PEB model. Second, because we are using approximate Bayesian inference, one can now accommodate nonlinear models, provided they are not too brittle (i.e., weakly non-linear). In other words, provided nonlinearities do not induce discontinuities in the free energy landscape. The implication is that when the model at the first level is linear, the approximate (variational) Bayesian inference scheme above becomes exact and one gets the same results that would be obtained using PEB, which itself generalises a classical ANCOVA. A numerical demonstration of this equivalence can be found in **DEMO_BMR_PEB.m**. Furthermore, the figures in this paper can be reproduced with **DEMO_DCM_PEB.m**, which can be regarded as equivalent demonstration for nonlinear models (and as a description of how to call the routines). In what follows, we examine the particular form of empirical Bayesian model reduction for group studies and then demonstrate its application to some simulated data in subsequent sections.

## Group studies with DCM

For DCM studies with *N* subjects and *M* parameters per DCM, we have a hierarchical model, where the responses of the *i*-th subject and the distribution of the parameters over subjects can be modelled as:

(11) $$\begin{array}{c} y_{i}=\Gamma_{i}^{\left(1\right)}\left(\theta^{\left(1\right)}\right)+\varepsilon_{i}^{\left(1\right)}\\ \theta^{\left(1\right)}=\Gamma^{\left(2\right)}\left(\theta^{\left(2\right)}\right)+\varepsilon^{\left(2\right)}\\ \theta^{\left(2\right)}=\eta +\varepsilon^{\left(3\right)} \end{array}$$

In this hierarchical form, *empirical priors* encoding second (between-subject) level effects place constraints on subject-specific parameters. The implicit generative model is defined in terms of multivariate Gaussian distributions (assuming the data for each subject are conditionally independent):

(12) $$\begin{array}{c} \ln p\left(y,\theta^{\left(1\right)},\theta^{\left(2\right)}|m\right)=\sum_{i}\ln p\left(y_{i}|\theta^{\left(1\right)}\right)+\ln p\left(\theta^{\left(1\right)}|\theta^{\left(2\right)}\right)+\ln p\left(\theta^{\left(2\right)}|m\right)\\ p\left(y_{i}|\theta^{\left(1\right)},m\right)=N\left(\Gamma_{i}^{\left(1\right)}\left(\theta^{\left(1\right)}\right),\Sigma_{i}^{\left(1\right)}\left(\theta^{\left(1\right)}\right)\right)\\ p\left(\theta^{\left(1\right)}|\theta^{\left(2\right)},m\right)=N\left(\Gamma^{\left(2\right)}\left(\theta^{\left(2\right)}\right),\Sigma^{\left(2\right)}\left(\theta^{\left(2\right)}\right)\right)\\ p\left(\theta^{\left(2\right)}|m\right)=N\left(\eta ,\Sigma \right) \end{array}$$

Generally, the second level would be a linear model where the random effects are parameterised in terms of their precision.

(13) $$\begin{array}{l} \Gamma^{\left(2\right)}\left(\theta^{\left(2\right)}\right)=\left(X⊗W\right)\beta \\ \Pi^{\left(2\right)}\left(\theta^{\left(2\right)}\right)=I_{N}⊗\left(Q_{0}+\sum_{j}e^{-\gamma_{j}}Q_{j}\right) \end{array}$$

Here, *β* ⊂ *θ* are group means or effects encoded by a design matrix with between *X* ∈ *ℝ*^*N* × *B*^ and within-subject *W* ∈ *ℝ*^*M* × *C*^ parts. The between-subject part encodes differences among subjects or covariates such as age, while the within-subject part specifies mixtures of parameters that show random effects. When every parameter can express different group effects: *W* = *I*_*M*_. Intuitively, the Kronecker product *X* ⊗ *W* models the fact that one or more parameters can show one or more group effects. We will assume that the first column of the design matrix is a constant term, modelling group means and subsequent columns encode group differences or covariates such as age. People familiar with linear models will notice that the model in Eq. (13) is effectively a (vectorised) multivariate linear model—something we will exploit later for classification.

The second (between-subject) level precision is parameterised by log precisions *γ* ⊂ *θ* of (positive definite) precision components *Q*_*j*_ that are added to a lower bound on precision *Q*_0_. It is these components that specify whether the parameters are random or fixed effects. Formally, the difference between a random and fixed effect rests upon the prior variance at the second level. Random effects have an informative prior that shrinks subject-specific estimates towards their (second level) mean. Conversely, fixed effects have a relatively flat or uninformative prior *Q*_0_ such that they are less influenced by parameter estimates from other subjects. Practically, the difference boils down to which parameters have nonzero precisions encoded by *Q*_*j*_ (in our software, random effects are specified in terms of the fields of Matlab parameter structures). One could estimate the empirical prior precision for every (random effects) parameter by specifying a large number of precision components. Alternatively, one can assume that the random effects are identically and independently distributed. In this paper, we will assume the latter, with a single precision component for all random effects.

We now wish to find the approximate posterior over second level parameters that maximises free energy, where the free energy at the first level has already been optimised for each subject under full priors. More precisely, we need the sufficient statistics of the approximate posterior $q\left(\theta^{\left(2\right)}|{\tilde{q}}^{\left(2\right)}\right)$, given the priors and approximate posteriors for each subject at the first level: $\left({\tilde{p}}_{i}^{\left(1\right)},{\tilde{q}}_{i}^{\left(1\right)}\right)$. From Eq. (10):

(14) $$\begin{array}{c} {\tilde{q}}^{\left(2\right)}=\left(\mu^{\left(2\right)},C^{\left(2\right)}\right)=\arg\max_{{\overset{⌢}{q}}^{\left(2\right)}}F^{\left(2\right)}\\ F^{\left(2\right)}=E_{q^{\left(2\right)}}\left[\sum_{i}F_{i}^{\left(1\right)}\left({\tilde{p}}_{i}\left(\theta^{\left(2\right)}\right)|{\tilde{p}}_{i}^{\left(1\right)},{\tilde{q}}_{i}^{\left(1\right)}\right)\right]-D_{KL}\left[q\left(\theta^{\left(2\right)}|{\tilde{q}}^{\left(2\right)}\right)||p\left(\theta^{\left(2\right)}|{\tilde{p}}^{\left(2\right)}\right)\right]\\ =\sum_{i}\left(F_{i}^{\left(1\right)}\left({\tilde{p}}_{i}\left({\tilde{q}}^{\left(2\right)}\right)|{\tilde{p}}_{i}^{\left(1\right)},{\tilde{q}}_{i}^{\left(1\right)}\right)+\frac{1}{2}tr\left(C^{\left(2\right)}\partial_{\mu^{\left(2\right)}}^{2}F_{i}^{\left(1\right)}\right)\right)-\frac{1}{2}{\mu^{\left(2\right)}}^{T}\Pi^{F}\mu^{\left(2\right)}+\frac{1}{2}\ln|\Pi^{F}C^{\left(2\right)}|\\ {\tilde{p}}_{i}=\left(\Gamma_{i}^{\left(2\right)}\left(\theta^{\left(2\right)}\right),\Sigma_{i}^{\left(2\right)}\left(\theta^{\left(2\right)}\right)\right) \end{array}$$

Note again that we only have to optimise the second level parameters, because the approximate posterior over the first level parameters are the reduced posteriors. At the second level, the conditional precision that maximises negative free energy is the solution to:

(15) $$\begin{array}{c} \partial_{C^{\left(2\right)}}F^{\left(2\right)}=\sum_{i}\frac{1}{2}\partial_{\mu^{\left(2\right)}}^{2}F_{i}^{\left(1\right)}+\frac{1}{2}P^{\left(2\right)}=0\Rightarrow \\ P^{\left(2\right)}=-\sum_{i}\partial_{\mu^{\left(2\right)}}^{2}F_{i}^{\left(1\right)}\Rightarrow \\ F^{\left(2\right)}=\sum_{i}F_{i}^{\left(1\right)}\left({\tilde{p}}_{i},{\tilde{q}}_{i}^{\left(1\right)}\right)-\frac{1}{2}{\mu^{\left(2\right)}}^{T}\Pi^{F}\mu^{\left(2\right)}+\frac{1}{2}\ln|\Pi^{F}C^{\left(2\right)}| \end{array}$$

In other words, the conditional precision is the sum of the (negative) curvature of the reduced free energy expected under the empirical priors. The second level free energy has the same form as in Eq. (10) but expressed in terms of reduced first level free energy summed over subjects. The second level expectations can now be optimised using gradient ascent as described in the Appendix. Note that the overall scheme can be applied recursively; in other words, once the second level parameters have been optimised they can be treated as first level parameters for efficient (recursive) inversion of deep hierarchical models.

In summary, we have an efficient scheme for the Bayesian inversion of (deep) hierarchical models that accommodate nonlinear and dynamical models at the first level. Note again that the summary statistics passed from one level to the next include not just the point estimators or expectations but the full (Gaussian) posterior density over parameters at the lower level.

### Bayesian classification

The classification of a new subject, in the context of hierarchical models, corresponds to evaluating the probability a new subject belongs to one group or another. Classification of this sort can be implemented efficiently using the sufficient statistics $\left({\tilde{\beta }}_{T},{\tilde{\gamma }}_{T}\right)$ of the empirical priors from a (training) group as full priors for the new (test) subject. This simply involves the Bayesian inversion of a hierarchical model of the new (test) data, in which the empirical priors are parameterised in terms of expected group effects and between-subject covariance:

(16) $$\begin{array}{c} \Gamma^{\left(2\right)}\left(\theta^{\left(2\right)}\right)=\left(\beta ⊗W\right){\tilde{\beta }}_{T}\\ \Sigma \left(\theta^{\left(2\right)}\right)=Q_{0}+\sum_{j}e^{-{{\overset{⌢}{\gamma }}_{T}}_{j}}Q_{j} \end{array}$$

Comparison with Eq. (13) shows that we have effectively swapped the roles of the explanatory variables in the design matrix and the model parameters. In other words, we are effectively estimating the class labels or covariates in the design matrix that best explain the response variables of the test subject. Clearly, some of these explanatory variables will be known; for example, the age of a subject. Similarly, if the first group effect is a group mean, then we know *β*_1_ = 1. Known values *β* ∈ *ℝ*^1 × *B*^ can therefore be fixed using appropriate priors (*η*, *Σ*), leaving unknown explanatory variables to be estimated based upon the test subject's posterior. This provides a Bayesian estimate of explanatory or diagnostic variables (e.g., class labels).

### Summary

In summary, we have a fairly straightforward set of expressions that enable inference under hierarchical models of (nonlinear) within-subject effects and (linear) between-subject effects. These can be applied in a number of different contexts. For example, we could simply assume that between-subject differences are attributable to random variations in their parameters and use a simple design matrix with *X* = **1** to provide empirical shrinkage priors, which shrink first level estimates towards the group mean in a Bayes-optimal fashion. We will consider this application in the next section. However, we can also consider more elaborate second level models that contain information about subjects and the groups from which they were sampled. This permits inference about group effects directly at the second or between-subject level or one can use Bayesian classification to estimate group membership using the empirical priors from a training group of subjects. We will consider this application in the last section.

## Empirical Bayesian model reduction—first level parameters

In what follows, we provide a worked example of how to apply Bayesian model reduction. This considers the analysis of a group study with DCM. Dynamic causal models are nonlinear state space models used to infer functional brain architectures and underlying effective connectivity. The parameters of these models include synaptic time constants and directed connection strengths. However, the details of the DCM used below are not important; in principle, the following can be applied to any DCM for any modality (e.g., functional MRI, EEG, MEG or local field potentials), or more generally to any nonlinear model. DCMs are generally nonlinear in two senses: first, the mappings between parameters and hidden states generating data are themselves nonlinear (for example, the sigmoid activation function in the DCM for evoked electromagnetic responses used below). Second, dynamic causal models require the integration of a dynamical system to generate a timeseries. This integration or solution is itself a nonlinear operation, introducing a further nonlinearity into the mapping from parameters to observations that would exist even in the absence of nonlinear dynamics.

Typically, when analysing group DCMs studies, people invert a series of models for each subject separately and then either harvest subject-wise parameter estimates from a selected model (identified as optimal at the group level) or use Bayesian model averaging to summarise each subject in terms of expected model parameters (Trujillo-Barreto et al., 2004, Penny et al., 2006, Woolrich et al., 2009). Note that the former is a special case of the latter, when the best model dominates the Bayesian model average. Generally, the Bayesian model average is preferred because it automatically accommodates uncertainty about the underlying model. The resulting subject-wise parameter estimates are then used as summary statistics for classical inference at the between-subject level (e.g., ANOVA or canonical variate analysis). In this section, we will focus on the use of Bayesian model reduction to provide more robust summary statistics for classical inference at the second level. In this application, there is no (potentially biassing) information about group differences in the second level design matrix, which consequently just models the group mean and any confounding variables, such as age or medication status. In short, our objective is to find the most efficient and robust way of estimating model parameters for subsequent classical inference.

**Fig. 1:**
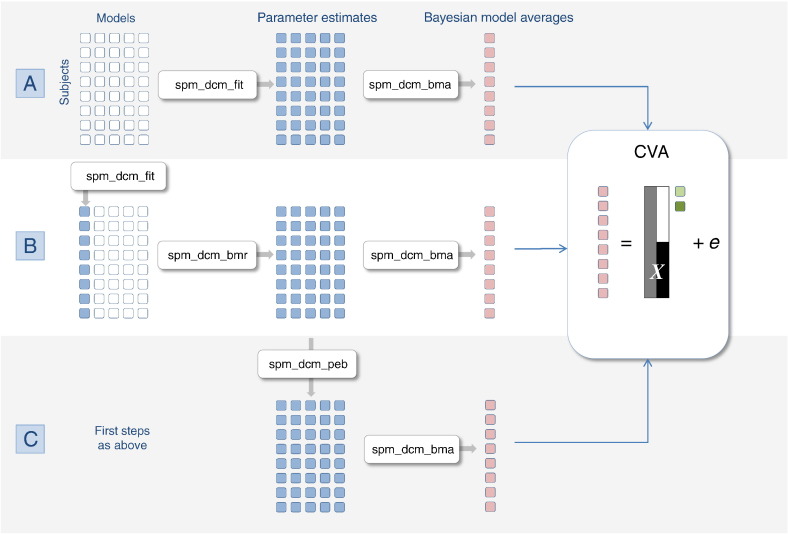
This figure provides a schematic overview of how various Bayesian procedures can be cast as operators on an array of models. The unfilled squares correspond to model specifications, while filled squares designate model structures that have been inverted or fitted to data to evaluate posterior densities over model parameters. Each column of the model array corresponds to a particular model (with the full or parent model in the first column). Conversely, each row of the array corresponds to a particular subject or dataset. First level parameter estimates are shown in blue and Bayesian model averages in red. This figure illustrates three ways that one could summarise subject-specific parameter estimates using Bayesian model averaging (i.e., accommodating uncertainty about which model generated the data). These three routes rest on various procedures (denoted here by their corresponding Matlab routine in the SPM software); namely, A. by direct inversion B. by inverting the full model (first column) and applying Bayesian model reduction or, finally, C. applying empirical shrinkage priors using empirical Bayes. CVA denotes canonical covariate analysis, which represents the standard way of making classical inferences about group effects, given a second level model in the form of a design matrix *X*.

To help organise the various procedures we have at our disposal, we will treat Bayesian model inversion, Bayesian model reduction, Bayesian parameter averaging, Bayesian model averaging and empirical Bayesian model reduction as operators on an array of model structures. This array is shown schematically in Fig. 1 as a matrix of unfilled squares that designate a set of models (in each row) of data from multiple subjects (in each column). Bayesian model inversion corresponds to evaluating the posterior density over model parameters (designated by a filled circle). This inversion can use any scheme, provided it returns the posterior expectations and covariance. In the SPM code, **spm_dcm_fit.m** automatically detects the nature of the model and applies the appropriate inversion scheme. Following inversion, one can then summarise the posterior density, either by averaging over models (in each row) or subjects (in each column). Bayesian model averaging (**spm_dcm_bma.m**) entails sampling from a mixture of posterior densities associated with each model that are, effectively, weighted according to the model likelihood. This provides a (non Gaussian) posterior density for each subject averaged over models. Conversely, Bayesian parameter averaging (**spm_dcm_bpa.m**) computes a posterior by effectively accumulating evidence over different subjects for the same model. This provides a (Gaussian) posterior density for each model averaged over subjects. In the context of group studies, Bayesian model averaging is generally used to provide subject-specific summary statistics. Bayesian model comparison (**spm_dcm_bmc.m**) assesses the evidence for each model, which is accumulated by simply summing the free energy over subjects (in each column). Alternatively, one might assume models are sampled at random, giving a random effects Bayesian model comparison (and subsequent Bayesian model averages). We will return to random model effects later.

Bayesian model reduction (**spm_dcm_bmr.m**) can now be considered as an alternative to Bayesian model inversion, provided the full model has been inverted. The schematic in Fig. 1 assumes that the full model occupies the first column. Finally, empirical Bayes (**spm_dcm_peb.m**) generates second level posteriors for each model that generally correspond to the group means and differences. These constitute empirical priors that shrink subject-wise estimates, thereby eliminating a degree of between subject variability. The second level posteriors are shown as green in Fig. 1, to distinguish them from the first level posteriors in blue.

Equipped with these operators, we can now ask how they could be best used to generate subject-specific summary statistics for subsequent (classical) inference. The first (and conventional) procedure would be to invert all the models for every subject and use Bayesian model averaging to generate parameter estimates for each subject. These Bayesian model averages have the advantage that they accommodate any uncertainty about the model. Alternatively, we could just invert the full model and use Bayesian model reduction, followed by Bayesian model averaging to produce summary statistics. Finally, we could apply empirical shrinkage priors by using empirical Bayes for each reduced model, under the assumption that each subject's parameters are random variations about the group mean. These three alternatives are shown in Fig. 1. What are the relative merits of these alternative procedures?

At first glance, it might seem that Bayesian model reduction should provide less robust estimates because it rests on the Laplace approximation, which may not hold for nonlinear models. In fact, based on the results presented here—and analyses of other data with different imaging modalities and data features, the opposite seems to be true: Bayesian model reduction appears to provide better estimators than the inversion of reduced models. This may be because Bayesian model reduction is less subject to the local maxima problem. In other words, inversion of the full model provides a higher dimensional parameter space that furnishes ‘escape routes’ from local maxima encountered during inversion in a low dimensional parameter space. This would clearly depend on the parameter space under evaluation, and to formally evaluate this conjecture one would need to use an approach that eschews the Laplace assumption, such as sampling (Sengupta et al., 2016). However, irrespective of the exact mechanism, Bayesian model reduction can be regarded as providing estimates of the posterior (and free energy) that would have been obtained if the Laplace approximation was true (for example, under linear models—see **DEMO_BMR_PEB.m** for a numerical illustration). The implicit robustness to violations of the Laplace assumption suggests that the reduced parameter estimates should be closer to the true values than the estimates following inversion of reduced models. To test this conjecture we simulated data using a fairly typical nonlinear (dynamic causal) model and compared the results of Bayesian model reduction to the inversion of reduced models, using the (true) parameters generating data as a reference.

### The simulated dataset

**Fig. 2:**
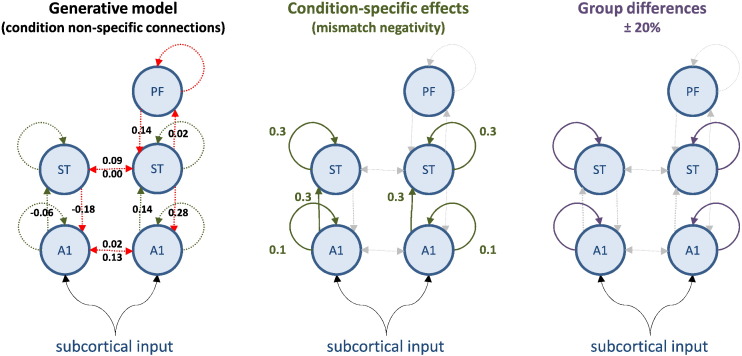
This figure describes the generative model used to simulated data for subsequent model inversion and reduction. The circles represent electromagnetic sources of data (recorded by 128 sensors or channels). These sources have intrinsic dynamics, modelled with eight ordinary differential equations per source. The dynamics are perturbed with a parameterised exogenous (subcortical) input and coupled to each other through (effective) connections. Because the sources are organised hierarchically, one can refer to (between-source or extrinsic) connections as forward or backward. The strengths of these connections correspond to the key model parameters with random effects. Left panel: The group average connection strengths (based on analysis of grand mean data from normal subjects) are shown alongside their connection: red connections do not change with experimental condition, while green connections are equipped with parameters encoding condition-specific effects. The values of the average connectivity over conditions are shown as log scale parameters. Middle panel: The strength of condition-specific differences (within subjects) are labelled in green. Again, these are log scale parameters, where a value of 0.1 corresponds roughly to a scaling of exp(0.1) = 1.1 or 10% increase in coupling. Right panel: The solid lines denote the condition-specific effects which differ between the two groups of eight subjects. These differences (plus or minus 20% about the average condition-specific effect) are restricted to intrinsic connections at the level of A1 and ST. A1—primary auditory source; ST—superior temporal source; PF—prefrontal source.

We chose a fairly complicated simulation setup to reproduce the sorts of data and questions typically encountered in DCM studies of clinical cohorts. We simulated two groups of eight subjects that can be regarded as normal and schizophrenic groups. We based the simulations on a previously reported EEG study of the mismatch negativity (Garrido et al., 2007)—a paradigm that has been modelled extensively using DCM in both normal subjects and schizophrenia (Garrido et al., 2009a, Garrido et al., 2009b, Fogelson et al., 2014). In brief, subjects are presented with streams of auditory tones, whose frequency is changed sporadically and unexpectedly. These correspond to *standard* and *oddball* stimuli, which evoke responses that can be recorded electromagnetically (here with EEG) as event related potentials. Previous studies have established a minimal network of five cortical sources that are sufficient to explain the form of these evoked responses, where differences between standard and oddball stimuli can be accounted for by differences in connectivity both within (intrinsic) and between (extrinsic) sources (Garrido et al., 2009a, Garrido et al., 2009b). See Fig. 2.

We generated data for each of the 16 subjects using the locations of five sources (right and left auditory sources, right and left superior temporal sources and a right prefrontal source) and the (connectivity) parameters estimated from a previously reported DCM study of the grand mean (Garrido et al., 2007). The grand mean estimates were used as group mean values, to which random Gaussian variates were added to produce subject-specific parameters. These random effects were sampled from the prior distribution over model parameters, described in Garrido et al., 2009a, Garrido et al., 2009b. More precisely, we fixed the between-subject parametric variability to be a sixteenth of the usual prior variances used in this sort of DCM. The usual prior variances now play the role of full priors on the second level parameters (the equivalent prior variance for second level precisions was set to unity and the prior expectations for both parameters and precisions was set to zero). In other words, we assumed that between-subject variability was smaller than our prior uncertainty about each parameter. This model has 158 neuronal parameters and 40 spatial parameters for each subject. Of these, we were particularly interested in 16 parameters encoding the strength of hierarchical connections between sources and how they changed with experimental condition (see Fig. 2).

We generated event related potentials using the lead field employed in the empirical study but with random (dipole orientation and amplitude) parameters sampled from their prior distribution. This introduced considerable variations in the evoked responses across subjects, in addition to between subject variability introduced by observation noise (see below). Crucially, we introduced systematic group differences in a subset of connections mediating differences in responses to standard and oddball stimuli; i.e., the mismatch negativity *per se*. In DCM, parameters modelling condition specific effects are generally encoded in a ‘B’ field, while an ‘A’ field encodes the (average) connectivity among the nodes of a network.

We inverted the grand mean data under a single known model that allowed for hierarchical connectivity among the five sources but restricted (within-subject) condition-specific effects generating the mismatch negativity to intrinsic and forward connections from the auditory and superior temporal sources. Crucially, between-subject differences were further restricted to changes in intrinsic connectivity (Kiebel et al., 2007, Fogelson et al., 2014). In other words, we are dealing with both within and between-subject differences in (nested) subsets of connections—subsets that we hoped to recover.

The values of condition-specific means and group-specific differences are provided in Fig. 2. The differences were chosen to be two standard deviations of the between-subject variance and modelled a systematic reduction in condition-specific effects in the second (schizophrenic) group. In other words, we simulated a failure or attenuation of the mismatch negativity in the last eight subjects that was restricted to intrinsic (within-source) connectivity changes in the auditory and temporal sources (these intrinsic connections generally involve inhibition of superficial pyramidal cells, which are the primary sources of observable signals). The values (between 0.1 and 0.4) of the condition-specific effects and group differences are moderate in quantitative terms. These values correspond to log scale parameters, in other words, a value of 0.1 corresponds roughly to a 10% increase.

**Fig. 3:**
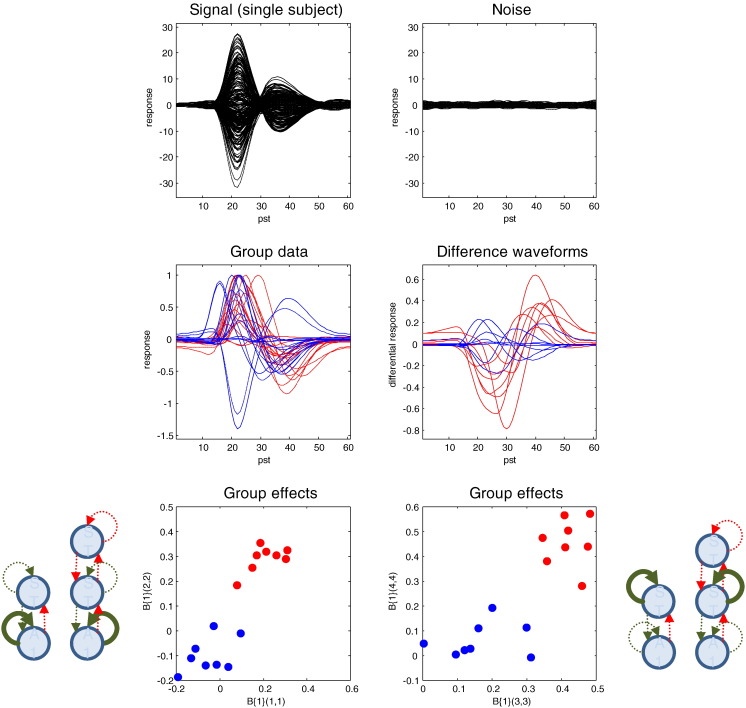
Simulated data. The upper panel shows channel data from a single subject as a function of peristimulus time in (4 ms) time bins. The solid lines (upper left) correspond to the simulated signal, while the dotted lines correspond to signal plus noise. For comparison, the observation noise is shown on the upper right. The middle panels show simulated responses over subjects in terms of a mixture of sensor data based upon the first principal component of the prior predictive covariance. The solid lines (middle left) correspond to the first (standard) condition, while the dotted lines report the second (oddball) condition for the first group of normal subjects (red lines) and second group of schizophrenics (blue lines). The middle right panel shows the condition-specific effects in terms of the waveform differences between the two conditions; namely, the mismatch negativity. It can be seen that this is markedly attenuated in the schizophrenic group. This attenuation is mediated by a reduction in the intrinsic connectivity enumerated in the previous figure. To illustrate the differences, in relation to between subject random effects, the lower panels plot the value of left and right intrinsic connection strengths for the first level of the auditory hierarchy (lower left) and the second level (lower right). It can be seen that these differences represent a fairly substantial effect size that we hoped to identify using Bayesian model reduction and empirical Bayes.

Fig. 3 shows an example of the data simulated over 128 channels. The upper left panel shows the simulated sensor data with and without simulated observation noise. Observation or sensor noise was created by convolving Gaussian noise with a smoothing kernel of eight (4 ms) time bins. The observation noise was scaled to have a standard deviation that was eight times smaller than the standard deviation of the simulated signal. This signal-to-noise ratio corresponds roughly to the levels that one would expect after averaging 64 trials, when the amplitudes of single-trial signal and noise are equivalent. The middle panels show the between-subject variance in terms of the response of the first principal component (of the prior covariance) over channel space; for the two conditions (left panel) and the condition-specific differences or mismatch negativity (right panel). These simulated responses illustrate the marked heterogeneity over subjects, largely induced by using random lead fields that map from neuronal responses to observed sensor data. Having said this, the effects of attenuating condition-specific changes in connectivity in the schizophrenic group are clearly visible in terms of impoverished mismatch negativities (blue traces), in relation to the normal group (red traces). The parameters generating these differences are shown in the lower panel and correspond to the subject-specific values of parameters encoding mismatch effects. In summary, we now have simulated data from two groups of subjects; where group differences lie not in connectivity *per se* but in the changes in (intrinsic) connectivity mediating condition-specific effects.

To illustrate the issues that attend Bayesian model comparison and averaging, we considered (first level) models that allowed condition-specific differences in forward connections, backward connections or intrinsic connections. All combinations of these three model factors create a space of eight models (Table 1). The condition-specific effects common to all subjects were generated under model three (forward and intrinsic changes), while the group differences corresponded to model four (intrinsic changes only). These differences are fairly subtle but not untypical and present a difficult inference problem, especially in the context of nonlinear modelling.

Finally, because we will be dealing with empirical Bayesian models, we also need to specify the second level model in terms of a design matrix. This comprised a constant term (first column) modelling a mean common to all subjects. The second column contained plus ones and minus ones, modelling a group effect between the first eight (normal) and second eight (schizophrenic) subjects. The final column of the design matrix contained random Gaussian variates and can be considered as modelling a confounding effect of age. We now use these data (and models) to illustrate the various approaches one can use to identify and quantify group differences.

### Bayesian model inversion, reduction or empirical Bayes?

We inverted the data from each subject using conventional Bayesian model inversion (Fig. 1A; **spm_dcm_fit.m**), Bayesian model reduction after inverting the full model without empirical reduction (Fig. 1B; **spm_dcm_bmr.m**) and with empirical reduction (Fig. 1C; **spm_dcm_peb.m**). The empirical Bayesian reduction assumed random effects on all the neuronal parameters and treated the measurement (lead field) parameters as fixed effects. In other words, we only applied shrinkage priors to the parameters of the presumed neuronal architecture generating data, allowing each subject to have different and unconstrained dipole orientations and magnitudes. Note that the second level model (design matrix) contained only a constant term to preclude any bias in subsequent tests for group differences.

**Fig. 4:**
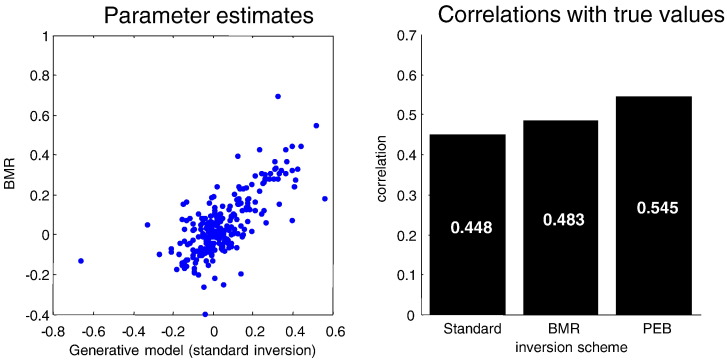
This figure reports the effects of Bayesian model reduction on the parameter estimates in terms of the 16 random effect parameters (connectivity and their condition-specific changes) by plotting the values obtained by Bayesian model reduction applied to the full model (y-axis, left panel) against those obtained by inverting the correct model without model reduction (x-axis). The quality of these estimates was assessed using their correlation with the true values, shown in the right hand panel for standard inversion of the generative model (standard), Bayesian model reduction (BMR) and subsequent empirical Bayes (PEB). The key result here is that one gets better estimates using Bayesian model reduction—and that these estimates further improve when applying empirical shrinkage priors.

Our first question was whether the quality of the parameter estimates, assessed with their correlation with true values, improved with Bayesian model reduction and subsequent shrinkage using empirical priors. If we had been dealing with linear models, the parameter estimates (expectations) following model reduction would not change. However, Fig. 4 shows marked differences between the estimates of the parameters under the true model using Bayesian model inversion and inversion of the reduced model. As intimated above, Bayesian model reduction provides better estimates in the sense that the correlation with the true values increases relative to the inversion of the true model (model three). Furthermore, the correlation increases again when we apply empirical Bayes. This provides a numerical confirmation of the conjecture that Bayesian model reduction is more robust to violations of the Laplace assumption. However, the robustness of Bayesian model reduction becomes even more important when considering Bayesian model averages.

### Bayesian model averaging

**Fig. 5:**
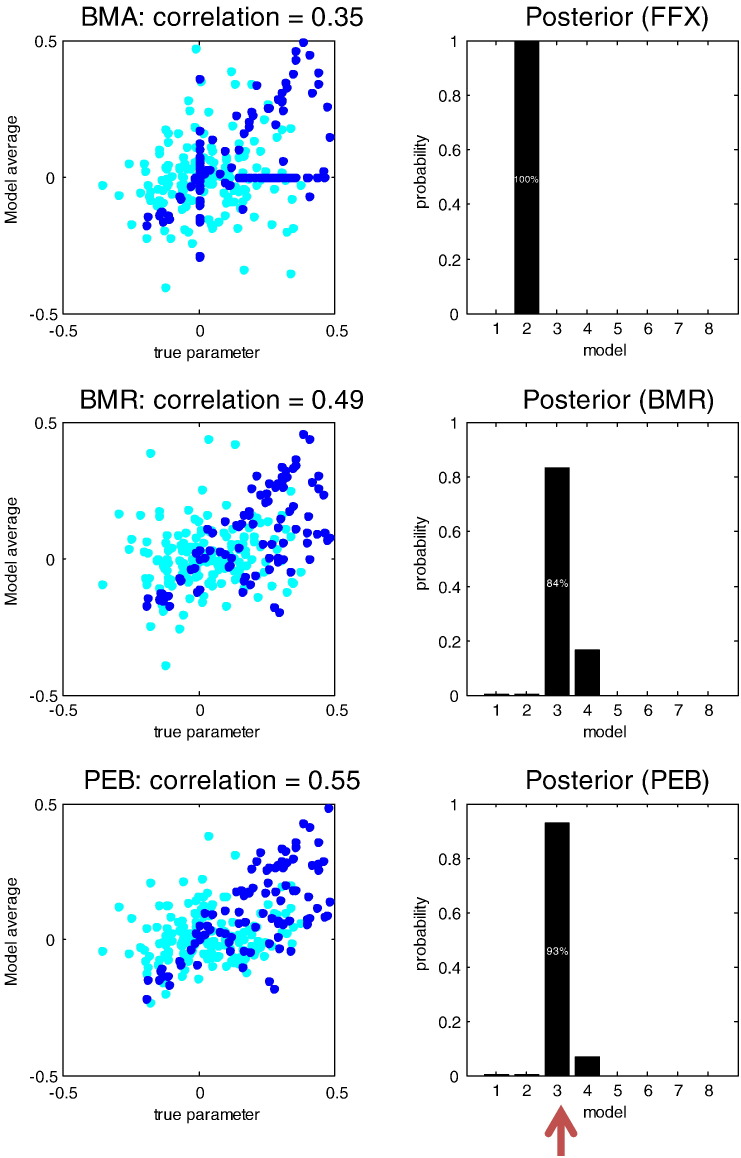
This figure (left panels) shows the improvement in the correlation between the estimates of connectivity (cyan dots) and their condition-specific changes (blue dots) with their true values, over all subjects. The right panels show the associated Bayesian model comparison in terms of model likelihoods over the eight (first level) models considered. The top row corresponds to a conventional inversion of the eight models (FFX). The middle row shows the equivalent results using Bayesian model reduction of the full model (BMR) and the last row shows the effects of applying empirical shrinkage priors with empirical Bayes (PEB). One can see the improvement in the correlations (the correlation coefficient is provided for each inversion scheme)—and the improvement in Bayesian model comparison: a standard scheme selects the wrong model (model two), whereas Bayesian model reduction correctly identifies the third model (red arrow), with increasing confidence (to over 90%) after empirical Bayes.

Bayesian model reduction also provides a reduced free energy that enables Bayesian model comparison and averaging. Using the eight models above, we computed the Bayesian model averages, weighting each model by its marginal likelihood pooled over subjects, for the three inversion schemes. In this case, the progressive improvement in the correlation with the true parameters is more marked. A fixed effects pooling of the free energy over subjects, under the standard inversion, incorrectly selects model two (Fig. 5, top row), presumably due to a violation of the Laplace assumption (e.g., local maxima). Whereas, the reduced free energy (Fig. 5, middle row) and the free energy under empirical Bayesian model reduction (Fig. 5, bottom row) correctly identify the third model. Model two has condition-specific modulation of the intrinsic connections, as per model three, but has modulation of top-down rather than bottom-up connections from superior temporal to primary auditory cortex. This means that the Bayesian model averages under the conventional scheme incorrectly assign (near) zero values to parameters that have nonzero values and *vice versa*. This is reflected in the vertical and horizontal rows of dots in the upper left panel of Fig. 5. Results of this sort suggest that it is important to use a robust scheme when using Bayesian model averages as summary statistics for subsequent inference about group effects.

**Fig. 6:**
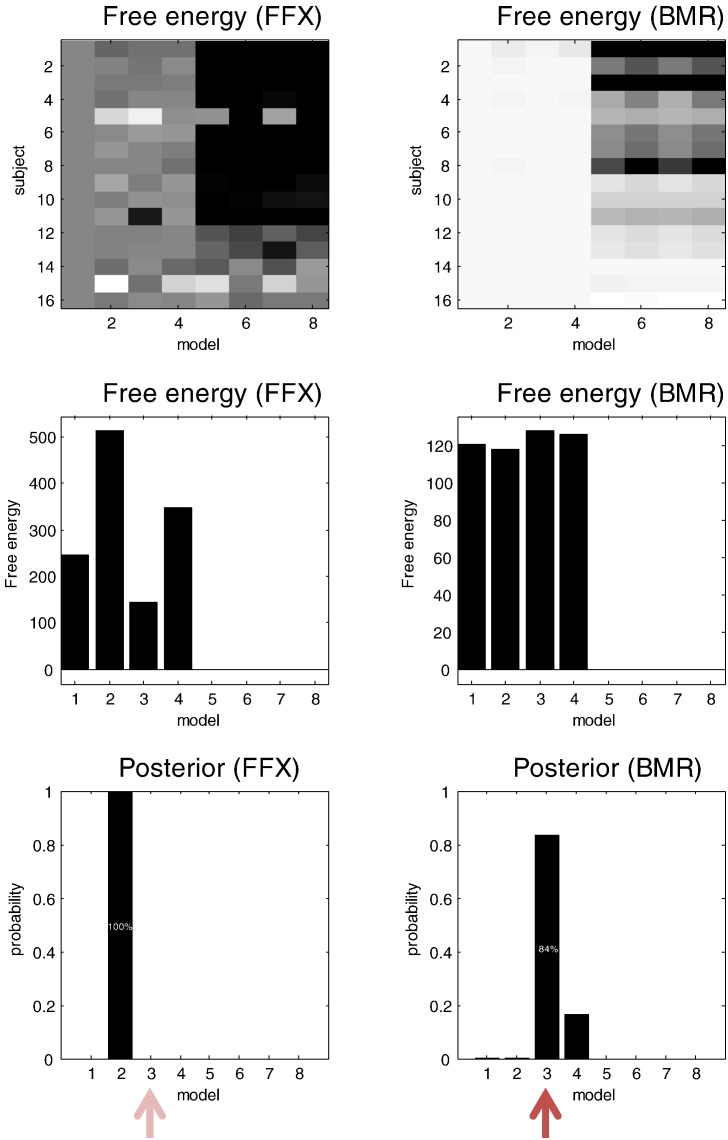
This figure illustrates the difference between inversion of reduced models and Bayesian model reduction on the (free energy) approximations to log model evidence—and ensuing model comparison. The left panels show the results for conventional inversion (FFX) and the right panels for Bayesian model reduction (BMR). The top row shows the free energies for every subject (rows) and model (columns). Here, we have used in Ockham's window of 512. In other words, we add a constant to the free energies so that the maximum is 512 (and then ignore free energies that are less than zero). Although the overall pattern of free energy is similar, the free energies under Bayesian model reduction are more stable (shown with an Ockham's window of 128 to highlight differences). The middle row shows the sum of free energies over subjects, while the lower row shows the corresponding model likelihoods, following an application of the softmax function to the free energies. Crucially, the correct model (red arrow) is only selected following Bayesian model reduction.

The panels of Fig. 6 show the accumulated free energies over the eight models under fixed effects assumptions. These are the sum of the free energies for each model over subjects, shown in Fig. 6 for the conventional inversion (left panels) and model reduction (right panels). The corresponding model likelihoods are shown in the lower panels (these correspond to posterior model probabilities under flat priors on models and are effectively a softmax function of the free energies). There are two key differences between the subject-specific free energies under conventional inversion and Bayesian model reduction: first, the differences under the conventional scheme are much greater than under Bayesian model reduction (note that we have used Ockham's windows of 512 and 128 when displaying the respective free energies—see the figure legend). Second, not only are the reduced free energies more stable over models, they have identified the correct model. It is also evident that changes in the intrinsic connections (first four models) are important explanations for the observed data. So does this mean that Bayesian model reduction provides a consistent profile of model evidence over subjects?

### Contribution of individuals to group effects

**Fig. 7:**
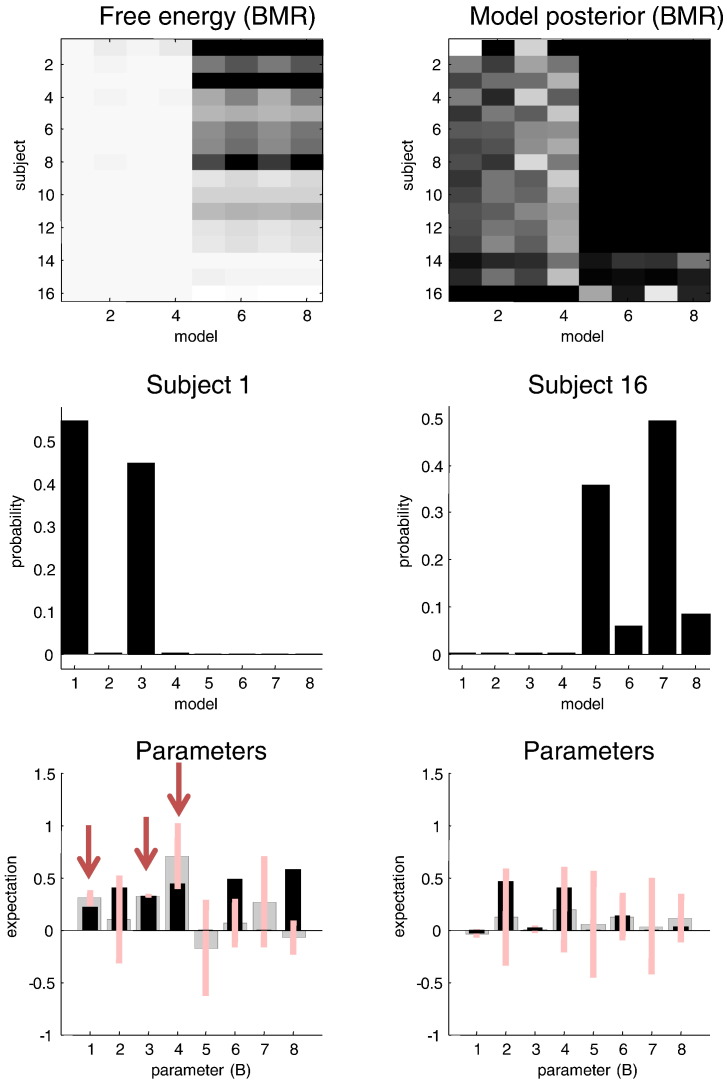
This figure provides a more detailed characterisation of model selection based upon free energies at the within-subject level. The upper left panel shows the free energies (following Bayesian model reduction) in Fig. 6. The corresponding model likelihoods for each subject are shown on the upper right. This illustrates a marked inconsistency in model selection over subjects, with some subjects supporting the full model and others the most reduced model. The lower panels show the model likelihoods for the first (left) and last subjects (right) who, by coincidence, have the greatest and least evidence for the full model. The (apparent) inconsistency can be understood in terms of the posterior densities over the key parameters that distinguish different models; namely, changes in connectivity. These densities are shown in terms of posterior means (grey bars) and 90% confidence intervals (pink bars) in the lower row. The key observation here is that only the first subject has posterior densities that exclude the prior mean and justify more complex models (red arrows). This is simply a reflection of the fact that the data from this subject provided more precise or efficient estimates.

Fig. 7 shows the same (reduced) free energies (upper left) as in the previous figure (using an Ockham's window of 128 to reveal greater detail) and the associated posterior probabilities for each subject (upper right). At first glance, these results are unsettling, because they show no consistent pattern: only three of the 16 subjects have the greatest evidence for the correct model. So what has gone wrong? In fact, nothing has gone wrong—the data from some subjects are better explained by a parsimonious model in which condition-specific effects cannot be detected in some connections. This is entirely expected given subject-specific differences in the way that data were generated; here, differences in the lead field mapping neuronal activity to sensory data. Heuristically, we are looking at the same responses but from different perspectives—the data from some subjects inform parameters of interest, while data from others do not. To emphasise this point, Fig. 7 provides the posterior estimates of the condition-specific changes for the subjects that have the greatest (lower left panels) and least (lower right panels) evidence for the full model. By coincidence, these were the first and last subjects, respectively. The key thing to note is that the first subject has posterior expectations of the parameters (defining the differences between models) that are clearly different from zero, whereas the last subject does not (the 90% Bayesian confidence intervals include the prior expectations of zero). This means the data from the last subject can be most parsimoniously explained with just changes in one or more forward connections (model seven). From the perspective of this subject there is no evidence for the correct model. The remaining parameters (which actually changed) are not sufficiently informed by this subject's data to justify the evidence for more complex models.

This example provides an unusual but tenable perspective on multi-subject studies using modalities like EEG. Effectively, we are hoping that one or more subjects disclose interesting parameters through the configuration of their cortical (electromagnetic) sources and sensor arrays; however, we fully anticipate that some subjects will not provide informative data for all parameters. Only when we tell the model that all the subjects were sampled from the same population and, implicitly, generate data under the same model, do we recover the global perspective inherent in studies of multiple subjects. Statistically, this requires a hierarchical or empirical Bayesian model. The shrinkage of parameter estimates for any given subject will be in proportion to how confident we are in the parameter estimates. In other words, subjects with uninformative data will be informed by subjects with informative data. This provides a heuristic explanation for the improvement in the quality of the parameter estimates (in relation to the true values) furnished by empirical Bayes.

### Random parameter or model effects?

**Fig. 8:**
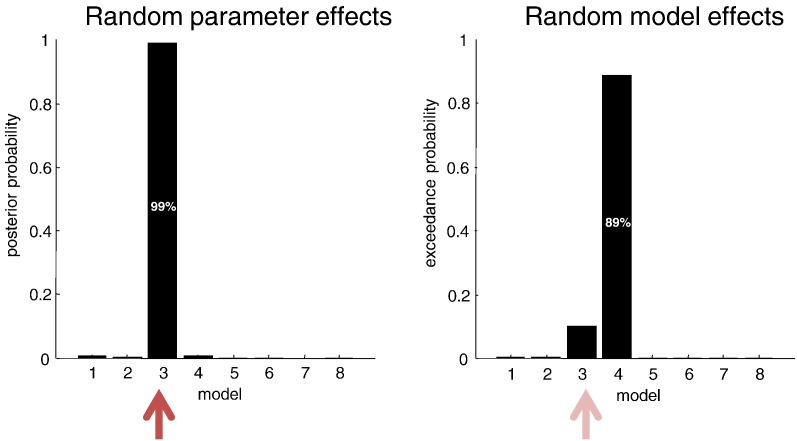
Model likelihoods based upon random parameter effects (left panel) and random model effects (right panel). In contrast to random model effects, Bayesian model reduction under parametric random effects selects the correct model (red arrows).

Unlike a conventional fixed effects model comparison, the underlying free energy of hierarchical models pertains to all the data from all subjects. In other words, there is only one (second level) free energy for each model. This can be compared with the free energy derived under random effects assumptions about models, as is commonly used for group DCM studies (Stephan et al., 2009). Fig. 8 shows the results of Bayesian model comparison in terms of model likelihoods, assuming random parametric effects (left panel) and random model effects (right panel). In this case, the correct random effects assumptions are parametric and, unlike the random model effect comparison, have identified (with more than 90% confidence) the correct model. Although the random model effects comparison identified the wrong model, the selected model only differs from the correct model by two connections—and is nearly right in a conservative direction. Indeed, this conservatism was one of the principle motivations for its introduction (Stephan et al., 2009).

With random parametric effects, we assume that between-subject differences are modelled in terms of random variations in the parameters under the same architecture or model, as opposed to random deletions or insertions of parameters (e.g., connections) implicit in random model effects. In the absence of (structural or pharmacological) lesions, the parametric random effects assumption, afforded by empirical Bayes is clearly more tenable. However, to disambiguate formally between random effects at the levels of parameters and models, one could use Bayesian model comparison—something we are currently working on.

### The summary statistic approach

**Fig. 9:**
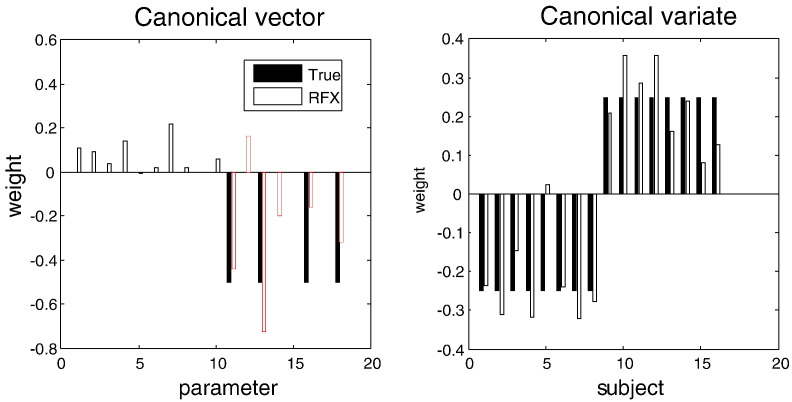
Principal canonical vector (left panel) and variate (right panel) following a canonical variate analysis of the Bayesian model averages (of connectivity and their changes) furnished by Bayesian model reduction and empirical Bayes. The true effects are plotted with filled bars and the corresponding vectors and variates with open bars. Both have been normalised so that their sum of squares is unity to help visualisation. The first 10 parameters in the left panel are the connections, while the last six (red outlines) correspond to condition specific changes in a subset of connections.

Having estimated the posterior densities over model parameters, one can now use their expectations as summary statistics for classical random effects modelling. In this example, our effects are multivariate and the appropriate (linear) inference scheme is a *canonical variates analysis* (aka multivariate linear regression, canonical correlation analysis, multivariate analysis of variance, Fisher discriminant analysis and, in limiting cases, Hotelling's *T*-square test). Subject-specific expectations of the average and condition-specific (connectivity) parameters were subject to canonical variates analysis testing specifically for group differences (as modelled in the between-subject design matrix). Fig. 9 shows the results of this analysis (**spm_cva.m**) in terms of the principal canonical vector (weights over parameters) and variate (weights over subjects). The canonical vector provides the weights of a mixture of parameters that shows the greatest correlation with a mixture of explanatory variables (i.e. group differences). Formally, this is equivalent to Hotelling's *T*-square test, having partialled out the constant term and age covariate (because there is only one remaining explanatory variable of interest). The result of this test was, as expected, exceedingly significant (*p* < 0.001).

More interestingly, the canonical variate does appear to reflect the group effect reliably (with the exception of the fourth subject). Furthermore, large canonical weights have been recovered for the four parameters (representing changes in intrinsic connectivity) that characterise group differences. At this point, one could report the results in terms of a classical *p*-value from the canonical covariates analysis and characterise the group effects quantitatively, in terms of the canonical vectors. This would have correctly identified the fact that the largest group effects are expressed in terms of attenuated intrinsic connectivity changes, underlying impoverished mismatch negativity responses in schizophrenia.

### Summary

This section has provided heuristics and numerical illustrations suggesting that Bayesian model reduction may be more robust to violations of the Laplace assumption during the inversion of nonlinear models. Furthermore, equipping models with hierarchical priors (empirical Bayes) optimally shrinks parameter estimates to a common mean; thereby providing more accurate estimates for subsequent analysis. In this section, we have illustrated the use of canonical variates analysis using the summary statistics afforded by Bayesian model reduction and empirical Bayesian inversion of (reduced) models. The opportunity to perform empirical Bayes with nonlinear models is potentially important because it allows us to consider random effects on parameters, as opposed to models. In the final section, we will see how much further we can take the empirical Bayesian approach in characterising group characteristics—and classifying new subjects.

## Empirical Bayesian model reduction—group effects

**Fig. 10:**
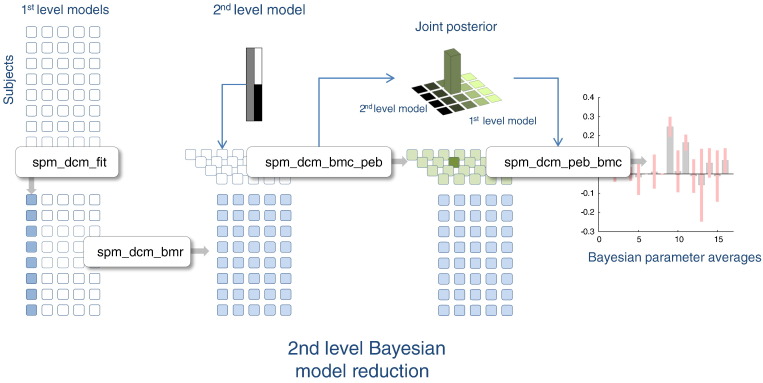
This schematic illustrates Bayesian model reduction and averaging at the second level. It follows the same format as Fig. 1 but focuses on the second level parameter estimates. After Bayesian model reduction, empirical Bayes can be used to estimate the posterior density over second level parameters for any combination of first and second level models. The winning model in this joint space can be further decomposed in terms of the combination of second level parameters contributing to group effects. After (second level) Bayesian model reduction, the ensuing Bayesian model averages can then be used to quantify between subject effects. In this paper, first level models correspond to different combinations of connection parameters (or their changes), while second level models correspond to different combinations of design matrix parameters, modelling between subject effects.

**Fig. 11:**
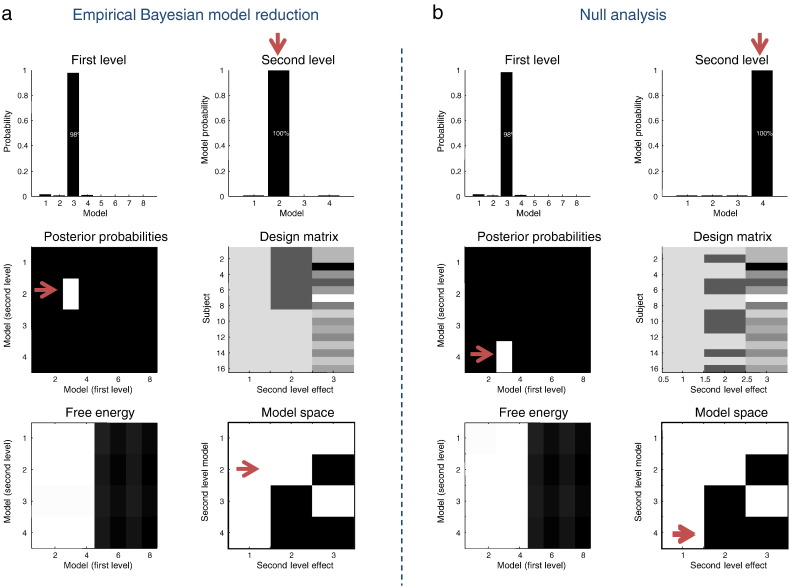
This figure illustrates the application of empirical Bayes to select the best model over the joint space of models at the first and second levels. (a) Results for the original setup. (b) The same results when randomising the diagnostic or group labels of each subject. This constitutes a null analysis. The top rows represent the marginal model likelihoods over the first level (left panels) and second level (right panels). Empirical Bayesian model reduction has correctly identified the third model at the first level and the second model at the second level. The latter model comprises the constant term and between-group differences and correctly discounts the covariate effect. These effects are encoded in the design matrices in the middle row, where different second level models correspond to different combinations of these effects (lower right panel). The underlying model likelihoods over the joint model space (and associated free energies) are shown in the lower left panels. Note that when we randomise diagnostic labels, empirical Bayesian model reduction (correctly) selects the model with no group differences (see red arrows).

In the world of empirical Bayes, there are models at each level of the hierarchy; here, first level models distinguish between different architectures at the level where data are generated and second level models contain explanatory variables at the between-subject level. This means that there is an implicit model space over the first and second levels. In turn, this affords the opportunity to perform Bayesian model comparison and averaging over the ensuing joint model space. In other words, for any given model at the first level there is a family of models at the second level that comprises all combinations of the explanatory variables (in the columns of the design matrix). We can now use empirical Bayesian reduction to score these models by using different combinations of explanatory variables for every first level model. The resulting free energies are now directly comparable because they are all functions of the same data. Practically, this entails a series of empirical Bayesian model reductions for each column of the reduced model array (**spm_dcm_bmc_peb.m**). See Fig. 10 for a schematic illustration. The results of this search of joint model space are shown in Fig. 11. In this section, we will restrict our analyses to the extrinsic connections that are common to all subjects and the condition-specific changes in (extrinsic and intrinsic) connectivity. In other words, we will treat the spatial parameters (and neuronal parameters that do not encode connectivity) as fixed effects.

Fig. 11a shows the design and results of this analysis. Second level effects (middle right panel) consisted of a constant term, group membership and age. These were combined into four models (lower right panel): model 1 included group and age, model 2 included group only, model 3 included age only and model 4 included neither group nor age. The marginal probabilities over first and second models are shown in the upper panels, demonstrating we can be 98% certain that the third (true) architecture generated our within-subjects data—and almost 100% certain that model 2 best explained group differences, correctly dispensing with the age covariate. For completeness, the corresponding posterior probabilities for each individual model are shown in the middle and lower left panels.

### Classical and Bayesian inference of the group level

To illustrate the fundamental difference between classical and Bayesian inference at the between-subject level, we then repeated the above procedure but randomly permuted the explanatory variables encoding diagnostic class. The results of this null analysis (Fig. 11b) indicate that we can be almost certain that the only between subject effect is a common mean. In other words, we can positively infer that there are no group effects. This inference would not be possible with classical inference. For example, had we failed to demonstrate a significant effect in the canonical covariates analysis above, we would only be able to say we had failed to reject the null hypothesis. This absence of evidence would not be evidence for the absence of group differences; simply that we had insufficient evidence. Conversely, with Bayesian inference we can accept the null hypothesis of no group differences provided our data are sufficiently informative. This is fundamentally different from saying we are not confident about the presence of group effects. If the data were extremely noisy or uninformative, then the model likelihoods would be uniformly distributed over all four second level models. In this case, we would have to think carefully about the quality of our data or experimental design. In this example, our data are sufficiently precise to preclude group effects, when the diagnostic labels have been deliberately randomised.

### Bayesian model comparison and averaging at the second level

So far, we know that there are group effects and these are expressed within a particular first level model; however, we do not know precisely which first level parameters (i.e. connections) subtend the group effect. This question calls for Bayesian model comparison and averaging at the second level (under the best model at the first level). The possible model space here can be quite complicated because (in the current setup) we have a second level parameter estimate for each second level explanatory variable. In other words, there is a pattern of parameters that encode different between subject effects such as group differences or age. In this worked example, we will take an exhaustive approach and simply score every possible combination of second level parameters, thereby considering all possible combinations of connections that show all possible combinations of group effects. This exhaustive search represents a third application of Bayesian model reduction in this paper. Here, we applied Bayesian model reduction to models at the first level (**spm_dcm_bmr.m**). We then applied the same technology to perform empirical Bayesian model reduction, considering every first level model (using **spm_dcm_bmc_peb.m**); although, one could also just estimate the second level effects under the full model using (**spm_dcm_peb.m**). In either case, one can now apply Bayesian model reduction to the posterior densities over the second level parameters (**spm_dcm_peb_bmc.m**) to find out where the key between-subject effects are expressed; in other words, identify the *β* parameters in Eq. (13) that mediate group effects.

**Fig. 12:**
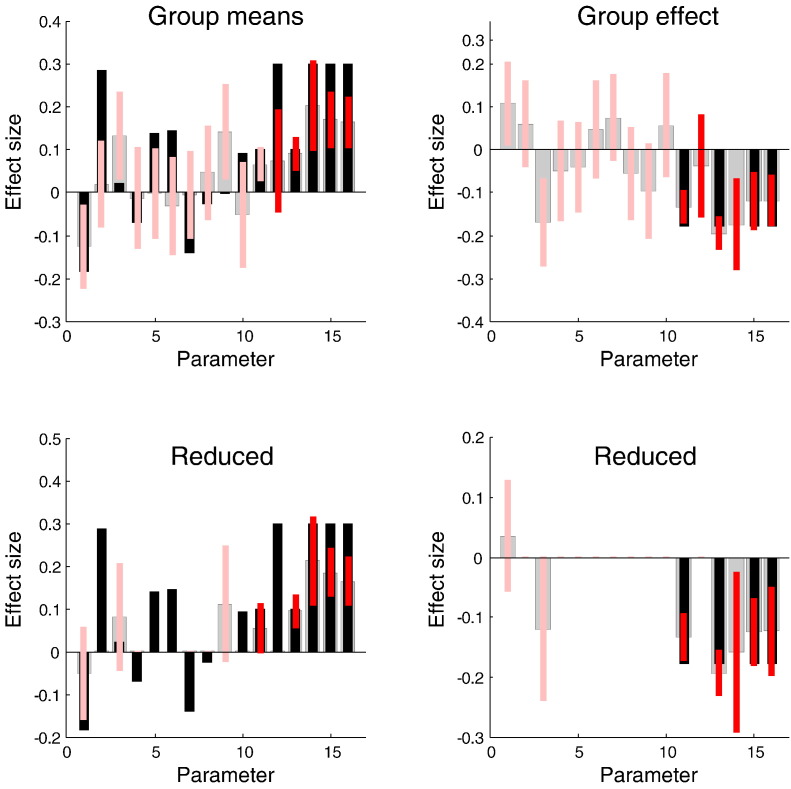
Bayesian model averages – under the best model identified in the previous figure – before (upper panels) and after (lower panels) Bayesian model reduction at the second level. These averages are shown using the same format as in previous figures, with the posterior means in grey and the 90% confidence intervals in pink. Black bars are the true values. Posterior densities are shown separately for the constant term or group means (left panels) and group differences (right panels). The connectivity parameters *per se* have pink confidence intervals, while the condition-specific changes (mismatch negativity effects) are shown with red confidence intervals. Note how Bayesian model reduction eliminates redundant parameters at the second level and has (largely correctly) identified the key group differences.

Fig. 12 shows the results of this second level Bayesian model comparison in terms of Bayesian model averages of the second level parameters. The upper panels show the posterior estimates before Bayesian model reduction and the lower panels after reduction. The key thing to observe is that some parameters have been removed. This automatic pruning of redundant parameters is the empirical Bayes equivalent of automatic relevance determination (Tipping, 2001). In this example, we see that all four changes in intrinsic connectivity – that characterise group differences – have been correctly identified (in the sense that the 90% Bayesian confidence intervals include, almost, the true values). Furthermore, Bayesian model reduction has correctly eliminated all but three parameters that showed no group differences. Although not a perfect result, it is comforting to note that the three parameters that should have been removed have the greatest posterior covariance (and two of them include the prior expectation within their 90% confidence interval). Before closing, we turn to another potentially useful application of empirical Bayes; namely, the prediction of class labels such as diagnostic categories.

### Bayesian classification and cross validation

We have portrayed empirical Bayes as supplying optimal shrinkage priors in the context of hierarchical model inversion. However, one can also treat the empirical priors as full priors when inverting a model of a new subject. In other words, one can use estimates of second level parameters (and between-subject precisions) from a group of *training* subjects to estimate unknown explanatory variables as described above. In our case, the interesting explanatory variable or class label is diagnostic (minus one for normal subjects and plus one for schizophrenics). Using Eq. (14), we can now evaluate the posterior beliefs about this explanatory variable for a *test* subject. To illustrate this procedure, we used a leave-one-out procedure. This involved empirical Bayesian model reduction of 15 subjects to compute the expected second level parameters (and precisions). These were then used to estimate the second explanatory (diagnostic) variable of the remaining subject, with tight priors on the first explanatory variable (i.e., constant term) and covariate (i.e., age).

**Fig. 13:**
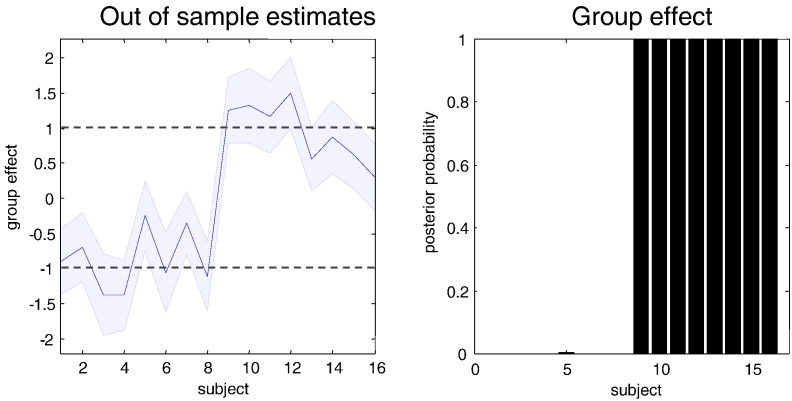
This figure shows the results of a leave-one-out analysis to illustrate Bayesian classification. The left panel shows the posterior predictive density over the group membership or diagnostic variable for each subject, based upon the second level parameter estimates obtained from the remaining subjects. These predictive densities can be converted into a diagnostic or classification probability (using Bayesian model reduction) to provide the probability that each subject belongs to the second (patient) group, shown in the right panel. In this case, every subject has been correctly classified; furthermore, this classification could be made with a posterior confidence of nearly 100%.

Fig. 13 shows the resulting predictive posterior densities over the diagnostic variable for every subject (using **spm_dcm_ppd.m**). This figure shows an almost complete separation of the 90% Bayesian confidence intervals with a reasonably accurate prediction of the true explanatory variable. This posterior density would be appropriate for continuous variables; however, our diagnostic variable is categorical. We can now appeal to Bayesian model reduction yet again to evaluate the evidence for models in which the diagnostic variable is exactly plus one, relative to models where it is exactly minus one. This simply involves setting the reduced expectation to the appropriate categorical value and the reduced covariance to zero. The reduced free energies then provide the probabilities of the categorical hypotheses (i.e. classifications). These categorical probabilities are shown in the right panel of Fig. 13 and illustrate that – with these data – we could predict the correct diagnosis with (almost) 100% confidence in all subjects. This is not terribly surprising given the large (unstandardised) effect sizes we used. What is remarkable is that we have been able to identify the informative parameters that enable us to use estimates of these effect sizes to perform classification.

The particular leave-one-out scheme used here (**spm_dcm_loo.m**) automatically performs Bayesian model reduction over a set of simple models (with point prior masses over unique values of the explanatory variable). This application of Bayesian model reduction to test simple hypotheses is formally equivalent to the Savage–Dickey ratio test (Savage, 1954). When there are more than two (simple) models, the resulting profile of model probabilities constitutes a (posterior predictive) probability distribution over the explanatory variable in question: e.g., over a discrete set of ages.

Clearly, one could use the probability of getting this sort of classification performance by chance as a cross validation of Bayesian inference based on the entire group. This is because the posterior predictive densities in Fig. 13 (left panel) are based upon parameters optimised on independent training data. However, in this example, such cross-validation would be redundant.

### Summary

In summary, this section has illustrated the use of Bayesian model reduction in scoring model spaces; both in the joint space of first and second level models and in the space induced by considering all combinations of parameters at the second level. Note that the second level parameters encode parametric effects for every between-subject effect, enabling us to find the best combination of parameters explaining commonalities and differences. At this point, one can now report the results using the posterior probability of group effects in the joint space of first and second level models—or by marginalising over all first level models (as shown in Fig. 11). Having established the significance of group effects, the underlying effect sizes can then be quantified in terms of posterior densities with, or without, searching over different combinations (as shown in Fig. 12). Finally, empirical Bayesian estimators of the sort considered in this section can be used for Bayesian classification, allowing a probabilistic statement about unknown explanatory or diagnostic variables, given a new subject. This may be particularly useful in establishing the predictive validity of dynamic causal models in a clinical setting.

## Relation to established procedures

This paper has introduced a general empirical Bayesian framework to model comparison at the group-level which differs from previous group-level Bayesian model selection approaches (Stephan et al., 2009, Penny et al., 2010, Rigoux et al., 2014) in a number of ways. The current approach benefits from the computational efficiency (and robustness) of Bayesian model reduction; allowing for an efficient scoring and averaging of large sets of nested models. Its empirical Bayesian nature exploits group-level information for regularising subject-specific parameter estimates and allows for model-based classification of individual subjects. These are potentially major advances, and we anticipate that for standard DCM studies these procedures will become the method of choice. Empirical Bayesian model reduction may also provide a more efficient form of generative embedding, in which parameter estimates from a generative model are used as features for discriminant classifiers (Brodersen et al., 2011, Raman et al., under review).

Having said this, there are several reasons why the standard approach of inverting each model separately and using the resulting model evidence for fixed or random effects Bayesian model selection may continue to play an important role in certain applications. First, Bayesian model reduction is restricted to comparing nested models; this prohibits a comparison of models which differ in the functional form of the likelihood; for example, comparisons of bilinear versus nonlinear DCMs for fMRI (Stephan et al., 2008), neural mass versus mean field models of electrophysiological data (Marreiros et al., 2010), or models of behavioural data that derive from formally distinct computational theories. Second, while the present analyses suggest a pleasing robustness of Bayesian model reduction to local extrema encountered during model inversion, it remains to be seen whether this will suffice for highly nonlinear models, such as conductance-based DCMs (Marreiros et al., 2010). In this instance, it will be useful to compare empirical Bayesian approaches based on posteriors estimated under the Laplace assumption and those based upon sampling schemes.

## Conclusion

In conclusion, this technical note has considered the use of Bayesian model reduction as an efficient way to explore model spaces at the within-subject level—and to finesse the computational burden of inverting hierarchical (empirical) Bayesian models of multiple subjects. We have touched upon a number of generic issues in Bayesian model comparison and random effects analyses; starting with the robustness of Bayesian model reduction when applying the Laplace approximation to nonlinear models and ending with the application of these procedures to Bayesian classification.

Based upon the heuristics and numerical behaviour of the Bayesian schemes we have considered, the following ‘standard practice’ for group DCM studies can be summarised as follows (see Fig. 10): first, specify a plausible (first level) model space, invert the full or parent model (**spm_dcm_fit.m**) and use Bayesian model reduction (**spm_dcm_bmr.m**). Then apply empirical shrinkage priors to the reduced models, using empirical Bayesian model reduction and a (second level) design matrix that precludes group differences (**spm_dcm_peb.m**). The ensuing Bayesian model averages (**spm_dcm_bma.m**) can then be used for classical inference with a design matrix that includes group effects (**spm_cva.m**).

Alternatively, one can pursue an empirical Bayesian analysis by using a design matrix with group effects. This begins, as above, with inverting the full model (**spm_dcm_fit.m**) and using Bayesian model reduction (**spm_dcm_bmr.m**). Then, the significance of the group effects (or their absence) can be established using Bayesian model reduction over the joint space of first and second level models (**spm_dcm_bmc_peb.m**). Finally, the Bayesian model averages of the second level parameters can be used to quantify and characterise group effects (with or without an exhaustive search over combinations of second level parameters, **spm_dcm_peb_bmc.m**). Although these procedures have yet to the applied extensively to real data, it will be interesting to see if they prove useful in a practical setting.

## Conflicts of interest

None

## Figures and Tables

**Table 1:** First-level model space. Models vary in whether their connections are modulated in the forward/bottom-up direction (forward), backward/top-down direction (backward) or in the self-connections (intrinsic).

Model	Condition-specific modulation
1	Intrinsic + forward + backward
2	Intrinsic + backward
3	Intrinsic + forward
4	Intrinsic
5	Forward + backward
6	Backward
7	Forward
8	None

## Appendix

The expectations of second level parameters in hierarchical models can be optimised using gradient ascent, where the gradients of (second level) variational free energy, with respect to the expected parameters at the second level $\mu^{\left(2\right)}=\left(\overset{⌢}{\beta },\overset{⌢}{\gamma }\right)$, are:

(A.1) $$\begin{array}{c} \partial_{\beta }F^{\left(2\right)}=\sum_{i}\partial_{\beta }F_{i}-\Pi_{\beta }\tilde{\beta }\\ \partial_{\beta \beta }F^{\left(2\right)}=\sum_{i}\partial_{\beta \beta }F_{i}-\Pi_{\beta }\\ \partial_{\beta }F_{i}^{\left(2\right)}=X_{i}^{T}\Pi_{R}\varepsilon \\ \partial_{\beta \beta }F_{i}^{\left(2\right)}=X_{i}^{T}\left(\Pi_{R}C_{R}\Pi_{R}-\Pi_{R}\right)X_{i}\\ X_{i}=X_{i\cdot }⊗W\\ \varepsilon =\mu_{R}-\eta_{R}\\ \eta_{R}=\Gamma_{i}\left(\mu^{\left(2\right)}\right)=X_{i}\tilde{\beta }\\ \Pi_{R}=\Pi_{i}\left(\mu^{\left(2\right)}\right)=Q_{0}+\sum_{j}e^{{\overset{⌢}{\gamma }}_{j}}Q_{j}\\ \mu_{R}=C_{R}\left(P_{i}\mu_{i}^{\left(1\right)}+\Pi_{R}\eta_{R}-\Pi_{i}^{\left(1\right)}\eta_{i}^{\left(1\right)}\right)\\ P_{R}=P_{i}^{\left(1\right)}+\Pi_{R}-\Pi_{i}^{\left(1\right)} \end{array}$$

These gradients are functions of the first level expectations and precisions of the *i*-th subject. Here, the sufficient statistics from the first level play the role of full priors (*η*_*i*_^(1)^, *Π*_*i*_^(1)^) and posteriors (*μ*_*i*_^(1)^, *P*_*i*_^(1)^), while the sufficient statistics from second level specify empirical priors (*η*_*R*_, *Π*_*R*_) that play the role of reduced priors. These empirical priors enable us to compute empirical Bayesian posteriors (*μ*_*R*_, *P*_*R*_) over parameters at the first level (see Eq. (8)). Note that the free energy gradient is zero when the average of the differences between the posterior expectations and the (empirical) prior is zero. The equivalent gradient and curvatures of the parameters of the empirical prior covariance are:

(A.2) $$\begin{array}{c} \partial_{\gamma }F^{\left(2\right)}=\sum_{i}\partial_{\gamma }F_{i}-\Pi_{\gamma }\overset{⌢}{\gamma }\\ \partial_{\gamma \gamma }F^{\left(2\right)}=\sum_{i}\partial_{\gamma \gamma }F_{i}-\Pi_{\gamma }\\ \partial_{\gamma_{j}}F_{i}^{\left(2\right)}=e^{{\overset{⌢}{\gamma }}_{j}}\frac{1}{2}\left(tr\left(\Sigma_{R}Q_{j}-C_{R}Q_{j}\right)-\varepsilon^{T}Q_{j}\varepsilon \right)\\ \partial_{\gamma_{j}\gamma_{k}}F_{i}^{\left(2\right)}=\left[j=k\right]\cdot \partial_{\gamma_{j}}F-e^{{\overset{⌢}{\gamma }}_{j}+{\overset{⌢}{\gamma }}_{k}}\left(\frac{1}{2}tr\left(\Sigma_{R}Q_{k}\Sigma_{R}Q_{j}-C_{R}Q_{k}C_{R}Q_{j}\right)-\varepsilon^{T}Q_{k}C_{R}Q_{j}\varepsilon \right) \end{array}$$

The Iversen brackets [*j* = *k*] return one when expression is true and zero otherwise. Note that the gradients depend upon the weighted sum of squared differences between the first level expectations and their empirical prior (c.f., the sample covariance subject-specific parameters). The second order derivatives with respect to parameters and precisions follow from the above:

(A.3) $$\begin{array}{c} \partial_{\beta \gamma }F^{\left(2\right)}=\sum_{i}\partial_{\beta \gamma }F_{i}\\ \partial_{\beta \gamma_{j}}F_{i}^{\left(2\right)}=e^{\gamma_{j}}{\left(X_{i}-C_{R}\Pi_{R}X_{i}\right)}^{T}Q_{j}\varepsilon \text{.} \end{array}$$

Finally, equipped with these gradients and curvatures we can now optimise the second level parameters using a regularised gradient ascent in the usual way (Ozaki, 1992):

*Until convergence*

(A.4) $$\begin{array}{c} \mu^{\left(2\right)}=\mu^{\left(2\right)}+\Delta \mu^{\left(2\right)}\\ \Delta \mu^{\left(2\right)}=\left[\begin{array}{c} \Delta \overset{⌢}{\beta }\\ \Delta \overset{⌢}{\gamma } \end{array}\right]=\left(\exp\left(\tau \cdot \partial^{2}F^{\left(2\right)}\right)-I\right){\left(\partial^{2}F^{\left(2\right)}\right)}^{-1}\cdot \left[\begin{array}{c} \partial_{\beta }F^{\left(2\right)}\\ \partial_{\gamma }F^{\left(2\right)} \end{array}\right]\\ \partial^{2}F^{\left(2\right)}=\left[\begin{array}{cc} \partial_{\beta \beta }F^{\left(2\right)} & \partial_{\beta \gamma }F^{\left(2\right)}\\ \partial_{\gamma \beta }F^{\left(2\right)} & \partial_{\gamma \gamma }F^{\left(2\right)} \end{array}\right]\\ q\left(\mu^{\left(2\right)}\right)=N\left(\mu^{\left(2\right)},-{\left(\partial^{2}F^{\left(2\right)}\right)}^{-1}\right) \end{array}$$

Here, *τ* is a regularisation parameter, which produces a conventional Newton scheme when large: note that the curvature of free energy ∂^2^*F*^(2)^ is negative, which means that the exponential term becomes an identity matrix when *τ* → ∞, in which case exp(*τ* ⋅ ∂^2^*F*^(2)^) → 0. These equations follow standard results using Variational Laplace; i.e., a gradient ascent on the variational free energy under the Laplace assumption. For full derivations please see (Friston et al., 2007).

## References

[bb0005] Bernal-Casas D., Balaguer-Ballester E., Gerchen M.F., Iglesias S., Walter H., Heinz A., Meyer-Lindenberg A., Stephan K.E., Kirsch P. (2012). Dynamical causal modeling reveals modulation of prefrontal–hippocampal connectivity by a genomewide significant schizophrenia variant. Front. Comput. Neurosci. Conference Abstract: Bernstein Conference 2012.

[bb0010] Brodersen K.H., Schofield T.M., Leff A.P., Ong C.S., Lomakina E.I., Buhmann M., Stephan K.E. (2011). Generative embedding for model-based classification of fMRI data”. PLoS Comput. Biol..

[bb0015] Efron B., Morris C. (1973). Stein's estimation rule and its competitors—an empirical Bayes approach. J. Am. Stat. Assoc..

[bb0020] Fogelson N., Litvak V., Peled A., Fernandez-del-Olmo M., Friston K. (2014). The functional anatomy of schizophrenia: a DCM study of predictive coding. Schizophr. Res..

[bb0025] Friston K. (2008). Hierarchical models in the brain. PLoS Comput. Biol..

[bb0035] Friston K., Penny W. (2011). Post hoc Bayesian model selection. NeuroImage.

[bb0030] Friston K., Mattout J., Trujillo-Barreto N., Ashburner J., Penny W. (2007). Variational free energy and the Laplace approximation. NeuroImage.

[bb0040] Friston K.J., Li B., Daunizeau J., Stephan K. (2011). Network discovery with DCM. NeuroImage.

[bb0045] Garrido M.I., Kilner J.M., Kiebel S.J., Friston K.J. (2007). Evoked brain responses are generated by feedback loops. Proc. Natl. Acad. Sci. U. S. A..

[bb0050] Garrido M.I., Kilner J.M., Kiebel S.J., Friston K.J. (2009). Dynamic causal modeling of the response to frequency deviants. J. Neurophysiol..

[bb0055] Garrido M.I., Kilner J.M., Kiebel S.J., Stephan K.E., Baldeweg T., Friston K.J. (2009). Repetition suppression and plasticity in the human brain. NeuroImage.

[bb0060] Kass R.E., Steffey D. (1989). Approximate Bayesian inference in conditionally independent hierarchical models (parametric empirical Bayes models). J. Am. Stat. Assoc..

[bb0065] Kiebel S.J., Garrido M.I., Friston K.J. (2007). Dynamic causal modelling of evoked responses: the role of intrinsic connections. NeuroImage.

[bb0070] Marreiros A.C., Kiebel S.J., Friston K.J. (2010). A dynamic causal model study of neuronal population dynamics. NeuroImage.

[bb0075] Ozaki T. (1992). A bridge between nonlinear time-series models and nonlinear stochastic dynamical systems: a local linearization approach. Stat. Sin..

[bb0080] Penny W., Mattout J., Trujillo-Barreto N. (2006). Bayesian model selection and averaging. Statistical Parametric Mapping: The Analysis of Functional Brain Images.

[bb0085] Penny W.D., Stephan K.E., Daunizeau J., Joao M., Friston K., Schofield T., Leff A.P. (2010). Comparing families of dynamic causal models. PLoS Comput. Biol..

[bb0090] Raman S.S., Deserno L., Schlagenhauf F., Stephan K.E. (2015). A Hierarchical Model for Unifying Unsupervised Generative Embedding and Empirical Bayes.

[bb0095] Rigoux L., Stephan K.E., Friston K.J., Daunizeau J. (2014). Bayesian model selection for group studies—revisited. NeuroImage.

[bb0100] Rosa M.J., Friston K., Penny W. (2012). Post-hoc selection of dynamic causal models. J. Neurosci. Methods.

[bb0105] Roweis S., Ghahramani Z. (1999). A unifying review of linear gaussian models. Neural Comput..

[bb0110] Savage L.J. (1954). The Foundations of Statistics.

[bb0115] Sengupta B., Friston K.J., Penny W.D. (2016). Gradient-based MCMC samplers for dynamic causal modelling. NeuroImage.

[bb0120] Stephan K.E., Kasper L., Harrison L.M., Daunizeau J., den Ouden H.E.M., Breakspear M., Friston K.J. (2008). Nonlinear dynamic causal models for fMRI. NeuroImage.

[bb0125] Stephan K.E., Penny W.D., Daunizeau J., Moran R.J., Friston K.J. (2009). Bayesian model selection for group studies. NeuroImage.

[bb0130] Tipping M. (2001). Sparse Bayesian learning and the relevance vector machine. J. Mach. Learn. Res..

[bb0135] Trujillo-Barreto N.J., Aubert-Vázquez E., Valdés-Sosa P.A. (2004). Bayesian model averaging in EEG/MEG imaging. NeuroImage.

[bb0140] Woolrich M.W., Jbabdi S., Patenaude B., Chappell M., Makni S., Behrens T., Beckmann C., Jenkinson M., Smith S.M. (2009). Bayesian analysis of neuroimaging data in FSL. NeuroImage.

